# Mapping the time-varying functional brain networks in response to naturalistic movie stimuli

**DOI:** 10.3389/fnins.2023.1199150

**Published:** 2023-06-16

**Authors:** Limei Song, Yudan Ren, Kexin Wang, Yuqing Hou, Jingsi Nie, Xiaowei He

**Affiliations:** ^1^School of Information Science and Technology, Northwest University, Xi’an, China; ^2^School of Foreign Studies, Xi’an Jiaotong University, Xi’an, China

**Keywords:** dynamic functional brain network, time-varying, dictionary learning and sparse coding, naturalistic stimuli, fMRI

## Abstract

One of human brain’s remarkable traits lies in its capacity to dynamically coordinate the activities of multiple brain regions or networks, adapting to an externally changing environment. Studying the dynamic functional brain networks (DFNs) and their role in perception, assessment, and action can significantly advance our comprehension of how the brain responds to patterns of sensory input. Movies provide a valuable tool for studying DFNs, as they offer a naturalistic paradigm that can evoke complex cognitive and emotional experiences through rich multimodal and dynamic stimuli. However, most previous research on DFNs have predominantly concentrated on the resting-state paradigm, investigating the topological structure of temporal dynamic brain networks generated *via* chosen templates. The dynamic spatial configurations of the functional networks elicited by naturalistic stimuli demand further exploration. In this study, we employed an unsupervised dictionary learning and sparse coding method combing with a sliding window strategy to map and quantify the dynamic spatial patterns of functional brain networks (FBNs) present in naturalistic functional magnetic resonance imaging (NfMRI) data, and further evaluated whether the temporal dynamics of distinct FBNs are aligned to the sensory, cognitive, and affective processes involved in the subjective perception of the movie. The results revealed that movie viewing can evoke complex FBNs, and these FBNs were time-varying with the movie storylines and were correlated with the movie annotations and the subjective ratings of viewing experience. The reliability of DFNs was also validated by assessing the Intra-class coefficient (ICC) among two scanning sessions under the same naturalistic paradigm with a three-month interval. Our findings offer novel insight into comprehending the dynamic properties of FBNs in response to naturalistic stimuli, which could potentially deepen our understanding of the neural mechanisms underlying the brain’s dynamic changes during the processing of visual and auditory stimuli.

## 1. Introduction

The study of functional brain networks (FBNs) can reveal the mechanisms and properties of brain functions, which is significant for elucidating the cognitive, sensory, and emotional functions of the brain (Rubinov and Sporns, 2010; Barrett and Satpute, 2013). The key characteristic of FBNs is their dynamic change across time for adapting to the continuously complex external environment (Hutchison et al., 2013; Calhoun et al., 2014; Ma et al., 2014; Lurie et al., 2020). Research on dynamic functional networks (DFNs) using functional magnetic resonance imaging (fMRI) have largely advanced our understanding of dynamic brain activity in responding to external sensory information (Tononi et al., 1996; Park and Friston, 2013; Calhoun et al., 2014).

Current studies on dynamic FBNs mainly rely on the resting-state paradigm (Hutchison et al., 2013; Allen et al., 2014; Liegeois et al., 2017; Savva et al., 2019). However, the resting-state is challenging to use when investigating specific cognitive processes due to its unrestrained nature and undesired behavioral disturbances, such as head movements and microsleep (Van Dijk et al., 2012; Buckner et al., 2013; Tagliazucchi and Laufs, 2014). In addition, electrophysiological and neuroimaging studies suggest that neural responses under the resting-state paradigm show general reliability and reproducibility (Belitski et al., 2008; Wang et al., 2017).

Naturalistic paradigms have been found to be more reliable and effective than the resting-state paradigm in exploring FBNs by providing cognitive constraints and high reliability (Sonkusare et al., 2019). These paradigms involve rich multimodal dynamic stimuli that reflect our everyday experience, resulting in more intricate patterns of functional brain activity and more diverse FBNs. Movies, as a typical representative of the passive viewing naturalistic paradigm, provide continuous audiovisual experiences that elicit stronger emotions than brief and isolated emotion-inducing events (Hasson et al., 2004; Meer et al., 2020; Saarimaki, 2021). Hence, using movies as stimuli in fMRI studies can better induce higher-order and complex FBNs related to cognition and emotion, thus leading to a more comprehensive understanding of DFNs and their relationship with cognition, sensation, and emotion.

However, most of the current research on DFNs focuses on the topology of time-varying connectivity, which limits the regions or nodes of the network to the selected template or the region of interest (ROI) (Hutchison et al., 2013; Calhoun et al., 2014). Less attention has been paid to the dynamic spatial patterns of the large-scale complex FBNs themselves induced by natural stimuli. To fully understand FBNs derived from fMRI data, it is necessary to investigate the spatio-temporal dynamics of these FBNs (Ge et al., 2020). In addition, while recent studies have analyzed the test–retest reliability of dynamic functional connectivity constrained by selected brain parcellation under the naturalistic paradigm (Tian et al., 2021; Zhang et al., 2021), the reliability of large-scale dynamic spatial patterns of FBNs remains unclear. Therefore, further research is required to explore the dynamic spatial patterns of FBNs and their relationship with cognition and perception, as well as their reliability during naturalistic conditions.

Inspired by the effectiveness of dictionary learning and sparse coding (DLSC) method in detecting static and dynamic FBNs (Lv, 2013; Ren et al., 2017a; Ge et al., 2020), we developed a data-driven method that combines group-wise DLSC approach with sliding window strategy, to identify and quantify the dynamic spatial patterns of time-varying FBNs from naturalistic fMRI data (NfMRI). Our method successfully identified several higher-order and complex FBNs, such as cerebellum-related networks, and revealed the significant correlations between movie annotations and detected DFNs. Additionally, we observed that specific individual DFNs were correlated with individuals’ subjective emotional perceptions to the movie. Furthermore, we validated the reliability of DFNs derived from two scanning sessions with 3 months intervals by evaluating their ICCs. In general, our study provides novel insights into the dynamic characteristics of FBNs under naturalistic stimuli.

## 2. Results

### 2.1. Group-wise static FBNs

**Figure 1:**
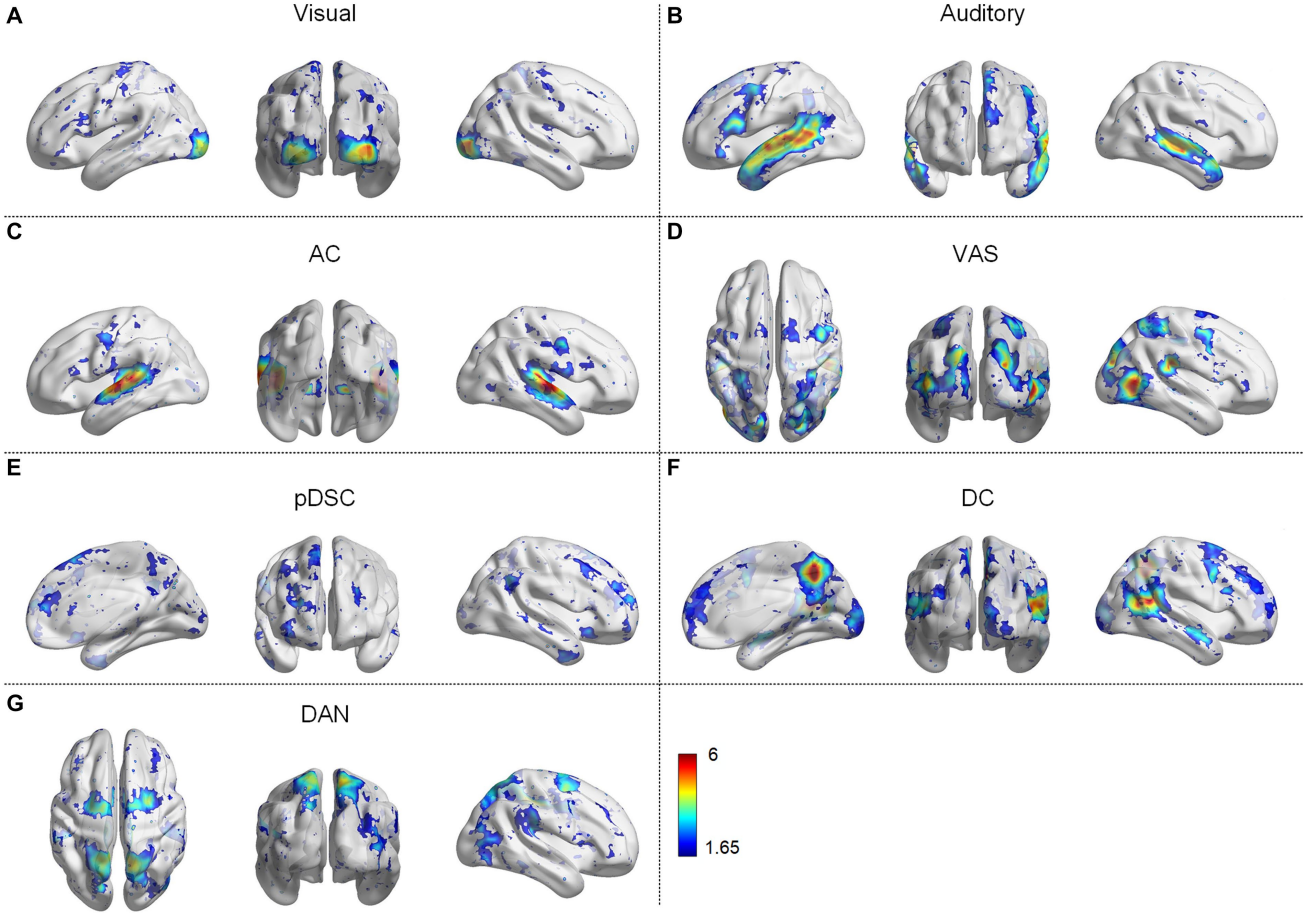
Group-wise static functional brain networks (FBNs) of session A, including **(A)** visual network, **(B)** auditory network, **(C)** auditory and cerebellar network (AC), **(D)** audiovisual and sensorimotor network (VAS), **(E)** partial default mode network (DMN), salience, and cerebellar network (pDSC), **(F)** DMN and cerebellar network (DC), **(G)** dorsal attention network (DAN).

We first identified seven consistent and representative group-wise static FBNs for both session A and session B via the DLSC approach. Figure 1 shows the representative FBNs of session A. These networks include either typically activated simple networks or complex networks. The simple networks involve the visual network (Figure 1A) and the auditory network (Figure 1B). The complex networks consist of multiple co-activated brain networks/regions, including auditory and cerebellar network (AC) (Figure 1C), the audiovisual and sensorimotor network (VAS) (Figure 1D), the partial default mode network (DMN), the salience and cerebellar network (pDSC) (Figure 1E), the DMN and cerebellar network (DC) (Figure 1F), and the dorsal attention network (DAN) (Figure 1G). Specifically, the AC network is primarily composed of auditory, cerebellar posterior crus 1,2 and vermis (Figure 1C). The VAS network is composed of visual, auditory, and sensorimotor cortex (Figure 1D). The pDSC encompasses the posterior cingulate cortex, medial prefrontal cortex, angular gyrus, anterior insula, dorsal anterior cingulate cortex, cerebellar posterior crus1,2, cerebellums 9 and vermis. Notably, the pDSC network excludes the precuneus (Figure 1E). The DC network mainly consists posterior cingulate cortex, medial prefrontal cortex angular gyrus, precuneus, cerebellar posterior crus1, 2, cerebellums 9 and vermis (Figure 1F). The DAN network includes intraparietal sulcus and the frontal eye fields (Figure 1G). A comparison between these identified FBNs and well-established resting-state templates or networks from previous studies conducted under natural stimulation is presented in Supplementary Figure S8.

The FBNs derived from session B showed a high degree of spatial consistency with those observed in session A (Supplementary Figure S1), as demonstrated by the relatively high overlap rate and Pearson correlation coefficient (PCC) values between the two sets of FBNs (Table 1). Specifically, the mean overlapping rate and the mean PCC of the seven FBNs were 0.44 ± 0.11 [Mean ± standard deviation (SD)] and 0.82 ± 0.18 (Mean ± SD), respectively, suggesting the consistency and stability of the DLSC framework in detecting FBNs across two scanning sessions.

**Table 1:** Overlapping rate and Pearson correlation coefficient (PCC) across two sessions for seven representative brain functional networks (FBNs).

	Visual	Auditory	AC	VAS	pDSC	DC	DAN	Mean ± SD
Overlap	0.34	0.58	0.5	0.52	0.27	0.5	0.39	0.44 ± 0.11
PCC	0.9	0.95	0.97	0.93	0.47	0.87	0.68	0.82 ± 0.18

### 2.2. Dynamic spatial patterns of FBNs

**Figure 2:**
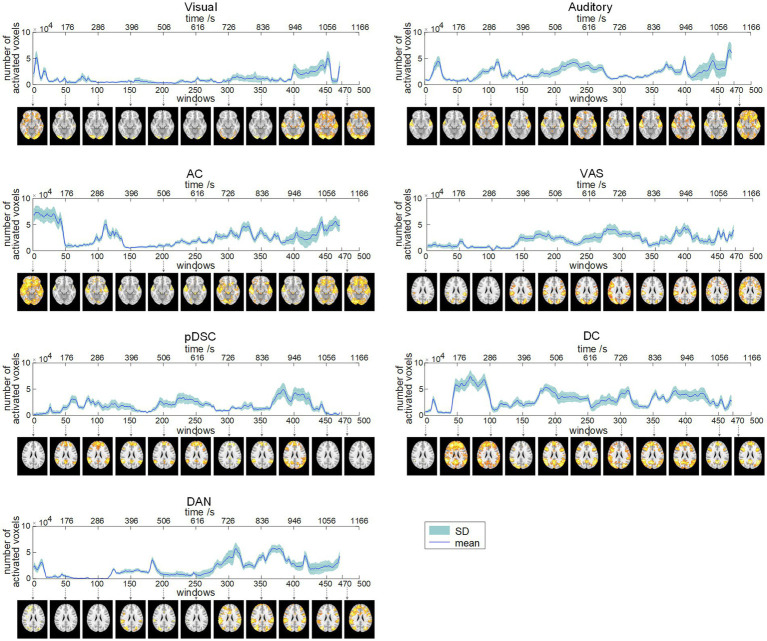
Dynamic evolution of the number of activated voxels (NAV) of seven brain function networks (FBNs) (session A). The corresponding FBNs of the first window among every 50 windows are displayed at the bottom.

We applied the sliding time window method with a window length of 60 repetition time (TR) units and step size of 1TR, resulting in 470 available windows. Correspondingly, 470 FBNs were obtained by applying the DLSC method, which could reflect the dynamics of time-varying large-scale networks. To provide representative visualization of these FBNs, we selected and displayed the FBNs from the first window among every 50 windows. For example, the first brain activation map in Figure 2 represents the visual network obtained during the first window (1TR to 60TR), corresponding to a time period of 1 s to 132 s.

**Figure 3:**
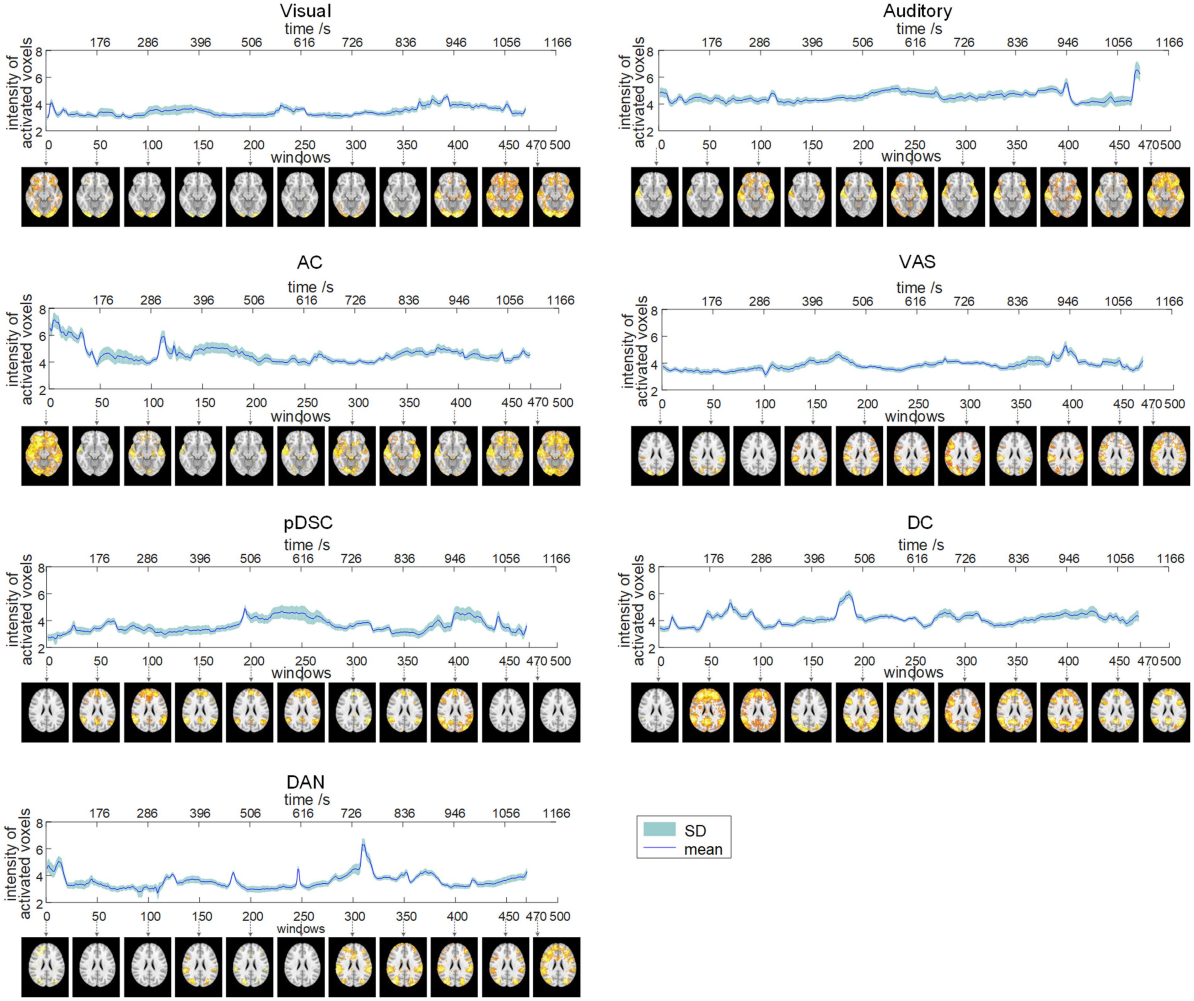
Dynamic evolution of the intensity of activated voxels (IAV) of seven FBNs (session A). The corresponding FBNs of the first window among every 50 windows are displayed at the bottom.

To quantitatively explore the dynamic spatial patterns of FBNs, we assessed the dynamic temporal changes of the number of activated voxels (NAV) (Figure 2) and the intensity of activated voxels (IAV) (Figure 3) for the seven FBNs. Our results revealed that for each network, both NAV and IAV exhibited temporal variations, with relatively consistent trends between the two metrics. The IAV showed less variability compared to NAV due to the calculation method of averaging activation intensities of all voxels exceeding a predefined threshold, resulting in a relatively narrow range of variation in the overall activation strength of the whole network. The peaks of NAV and IAV curves corresponded to the FBNs that displayed more pronounced and widespread patterns of activation, whereas the troughs of these curves responded to FBNs with diminished or even absent activation patterns. These findings highlight that FBNs were dynamic and evolved temporally in response to the unfolding plot of the movie, which is also consistent with the underlying neural basis of complex perception and behavior (Calhoun et al., 2014). Additionally, the lower-order perceptual networks, including visual network, auditory network, and VAS network, exhibited relatively stable level of activation over time, whereas the higher-order networks, such as pDSC, DC, and DAN networks, showed greater fluctuations in activation curves. The AC network, specifically, comprising both lower-order network (i.e., auditory network) and higher-order networks (i.e., cerebellar network), also displayed substantial fluctuations in its activation curves (Figures 2, 3). These results suggest that different FBNs exhibit distinct temporal dynamics in response to external stimuli, which may reflect their respective roles in higher-level cognitive and attentional processes.

The results of session B were generally consistent with those of session A, as evidenced by the visual comparison of the results depicted in Supplementary Figures S2, S3. Moreover, the PCC values for both NAV and IAV curves across session A and session B were relatively high for most FBNs (Supplementary Table S1), suggesting that the identified dynamic spatial patterns of FBNs induced by the movie viewing are reproducible and consistent across two scanning sessions.

### 2.3. Correlation between DFNs and movie annotations

To investigate the relationship between DFNs and the unfolding of the movie, we assessed the spearman correlation between dynamic changes of NAV/IAV and movie annotations, which include language use, changepoints, the presence of positive valence of scenes (scenes_p), the presence of negative valence of scenes (scenes_n), the presence of faces with positive (face_p), and presence of faces with negative (face_n). The results showed that two DMN-related networks, i.e., the DC and pDSC networks, were significantly correlated with movie annotations. Specifically, both NVA and IVA metrics of the DC network showed statistically significant correlations with the appearance of positive facial expressions based on permutation-based testing (*p* < 0.05) (see Methods) (Tables 2, 3). Notably, the value of p for the IAV metric was less than 0.01 (Table 3). Additionally, the changes in INV of the pDSC network were significantly and positively correlated with the appearance of the changepoint in the movie scenes (permutation 5,000 times, *p* < 0.05) (Table 3).

**Table 2:** The Spearman correlation between the changes in the number of active voxels (NAV) and movie annotations.

	Visual	Auditory	AC	VAS	pDSC	DC	DAN
language	−0.03	0.00	−0.09	−0.12	−0.17	−0.11	−0.14
changepoint	−0.15	−0.10	−0.04	0.11	0.02	0.14	0.06
scenes_p	0.00	−0.11	0.07	0.03	−0.05	0.08	−0.11
scenes_n	−0.10	0.01	−0.09	−0.19	−0.08	0.02	−0.12
face_p	−0.07	−0.11	0.08	0.00	−0.02	0.16*	−0.09
face_n	−0.05	0.02	−0.08	−0.28	−0.07	−0.01	−0.20

Bold font indicates significant correlation (*p < 0.05). Permutation test with 5,000 iterations.

**Table 3:** The Spearman correlation between the changes in the intensity of active voxels (IAV) and movie annotations.

	Visual	Auditory	AC	VAS	pDSC	DC	DAN
language	−0.14	0.03	−0.12	0.00	−0.17	−0.08	−0.08
changepoint	−0.18	−0.14	0.02	0.01	0.15*	0.12	−0.01
scenes_p	−0.18	−0.04	0.03	−0.10	0.04	0.13	−0.07
scenes_n	−0.01	0.07	−0.08	−0.06	−0.15	−0.01	−0.07
face_p	−0.06	−0.01	−0.02	−0.09	0.05	0.19**	−0.07
face_n	−0.12	0.04	−0.07	−0.19	−0.19	−0.06	−0.11

Bold font indicates significant correlation (*p < 0.05, **p < 0.01). Permutation test with 5,000 iterations.

### 2.4. Dynamic inter-subject correlation analyses

**Figure 4:**
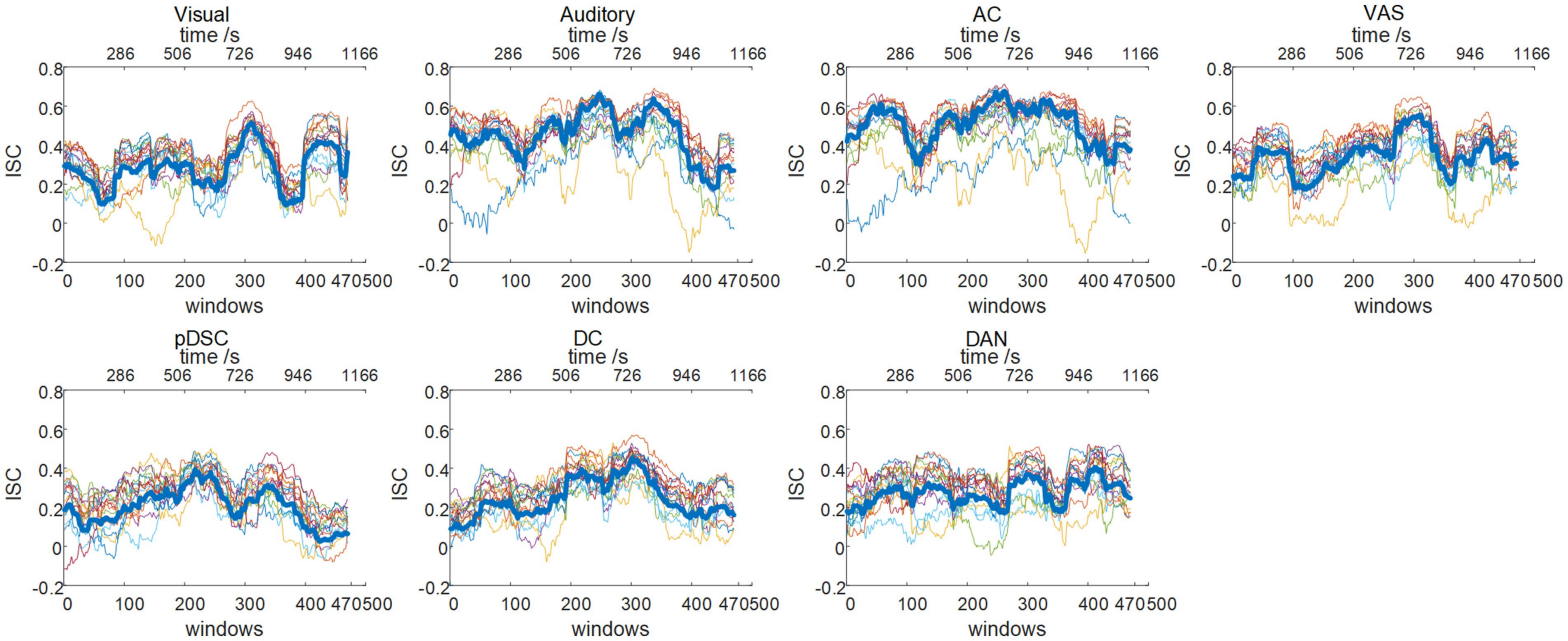
Dynamic inter-subject correlation (ISC) (session A): group-wise and individual dynamic ISC. The thick blue line represents the group-wise dynamic ISC, and the thin colorful lines depict the dynamic ISC of 16 different individuals.

The neural response evoked by the naturalistic stimuli exhibit not only high consistency across individuals, but also inter-subject variability and uniqueness reflecting personal experiences and intrinsically-driven processes under natural viewing condition, which varies across different brain regions/networks (Golland et al., 2007; Ren et al., 2017b). Hence, to quantify these group consistency and individual variations in defined DFNs, we adopted their corresponding group-wise static FBNs as templates to calculate the dynamic inter-subject correlation (ISC) (see Methods). Accordingly, the group-level dynamic ISC can represent the degree of temporal consistency across subjects in different FBNs (the thick blue line in Figure 4). The average values of group-level dynamic ISC during the entire period for seven FNBs (including visual, auditory, AC, VAS, pDSC, DC, and DAN networks) were 0.33 ± 0.12, 0.51 ± 0.13, 0.58 ± 0.12, 0.39 ± 0.12, 0.25 ± 0.10, 0.31 ± 0.08, and 0.34 ± 0.08 (Mean ± SD), respectively. While relatively high ISC values were observed in networks encompassing lower-level perceptual regions, especially those related to auditory processing, such as auditory and AC networks, the higher-order networks demonstrated lower ISC values that can indicate the occurrence of intrinsically-driven processes during individual movie viewing, including pDSC and DC networks, consistent with previous research (Ren et al., 2017b). Moreover, individual-level dynamic ISC also showed inter-subject variations especially in those higher-order networks under movie stimuli (colorful thin lines in Figure 4).

The dynamic ISC of session B were largely consistent with session A (Supplementary Figure S4). Quantitatively, the PCC values for seven FBNs across two sessions were relatively high (Table 4), with an average PCC value of 0.82 ± 0.18 (Mean ± SD), thereby reaffirming the consistency of DFNs identified by our DLSC framework across two sessions.

**Table 4:** The PCC of group-wise dynamic inter-subject correlation (ISC) across two sessions.

	Visual	Auditory	AC	VAS	pDSC	DC	DAN	Mean ± SD
PCC	0.89	0.63	0.87	0.79	0.76	0.86	0.83	0.81 ± 0.09

### 2.5. Correlations between movie ratings and individual differences in dynamic ISC of DFNs

**Figure 5:**
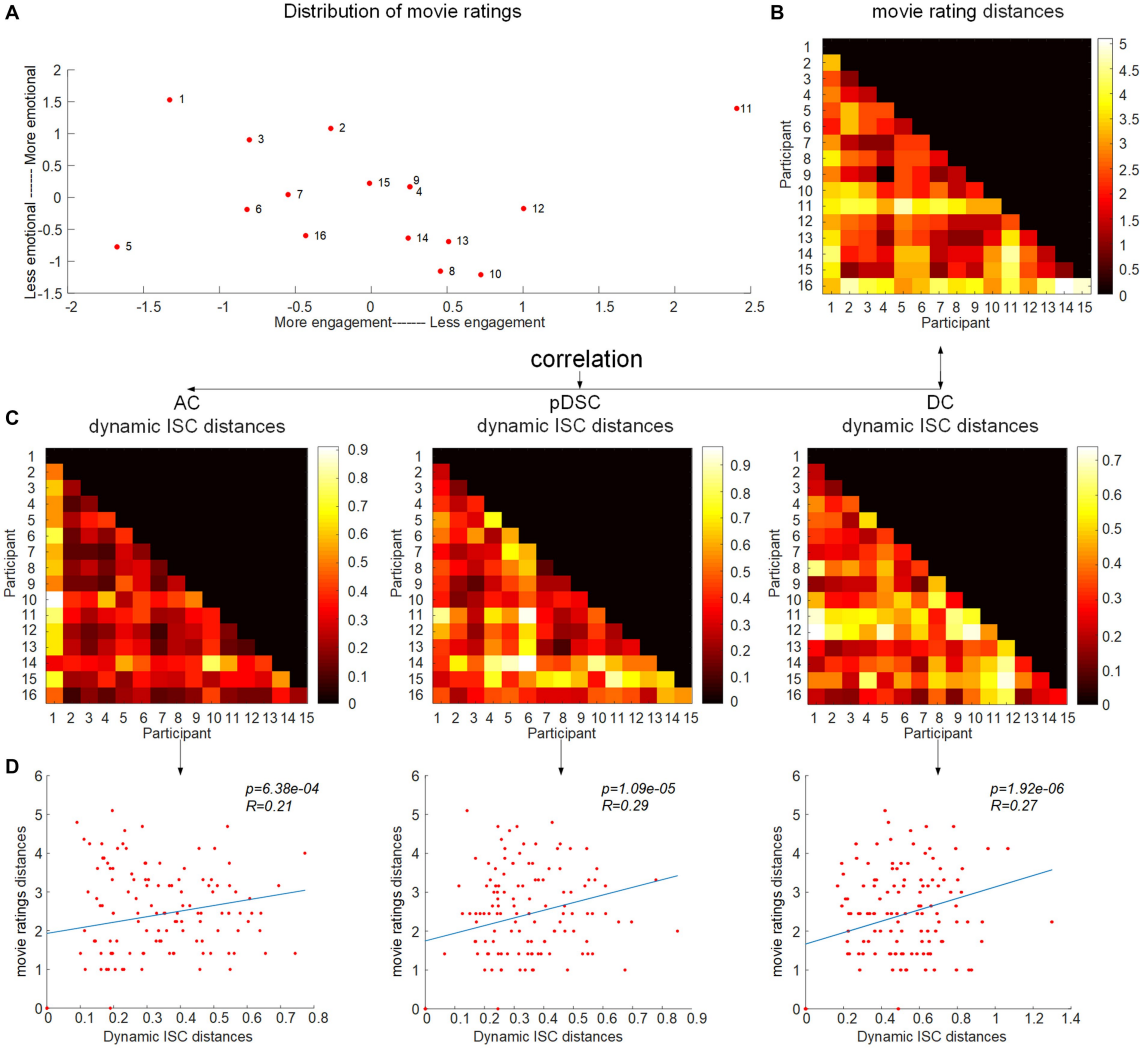
Correlation between the movie ratings and difference of individual dynamic ISC. **(A)** The inter-subject distances of the movie ratings were mapped onto a two-dimensional plane, with movie ratings shown in the inset and coded accordingly. The arrangement of movie ratings from left to right signifies participants’ engagement with the movie, as those who were more engaged reported higher levels of enjoyment, emotion, and audio quality and lower levels of boredom. The top-to-bottom scale reflects the participants’ ratings of evoked emotions. **(B)** Inter-subject distance matrix of the movie ratings. **(C)** Distance matrices of dynamic ISC for AC, pDSC, and DC networks. **(D)** The correlation between the movie rating distances and the inter-subject distances of dynamic ISC.

In the preceding section, there was relatively lower consistency in individual neural responses observed in higher-order brain networks, such as the pDSC and DC networks. This variability may be indicative of individual differences and unique experiences during natural viewing conditions. To investigate this assumption further, that is, exploring the potential relationship between subjective movie viewing experiences and the dynamics of DFNs, we examined whether the individual dynamic ISCs were correlated with their personal ratings of the movie. We applied an inter-subject representational similarity analysis (IS-RSA) (see Methods) to explore whether participants with similar subjective ratings also exhibited similar dynamic neural response patterns. Specifically, we employed a multidimensional scaling approach (MDS) (Carroll and Arabie, 1998) to characterize the answers to the post-movie questionnaire. Our result revealed that participants had varying experiences while watching the movie, with some reporting high engagement characterized by low boredom, high enjoyment, high emotion, and high audio quality, while others had low engagement (Figure 5A). The distances between movie ratings were measured by the Euclidean distance matrix of questionnaire answers across all individuals (Figure 5B). We computed Pearson distance to represent the inter-subject distances of dynamic ISC values for seven representative FBNs, respectively (Supplementary Figure S5). By evaluating the correlation between the movie rating distances and the inter-subject distances of the dynamic ISC, we found significant positive correlations (permutation 5,000 times, *p* < 0.05) in three cerebellum-related networks, that is, AC, pDSC, and DC networks. The distance matrices of dynamic ISC for these three networks are presented in Figure 5C, and their simple linear regressions are shown in Figure 5D. However, the other DFNs did not show statistically significant associations (*p* > 0.05) (Table 5). We did not repeat this experiment in session B as it involved a repeated viewing of the same movie, and the post-viewing questionnaire was not conducted.

**Table 5:** The Spearman correlation between movie rating distances and inter-subject distances of dynamic ISC for seven representative FBNs.

	Visual	Auditory	AC	VAS	pDSC	DC	DAN
Correlation	−0.02	0.07	0.15*	0.13	0.20*	0.17*	0.05

Bold font indicates significant correlation (*p < 0.05). Permutation test with 5,000 iterations.

### 2.6. Test–retest reliability of DFNs

**Figure 6:**
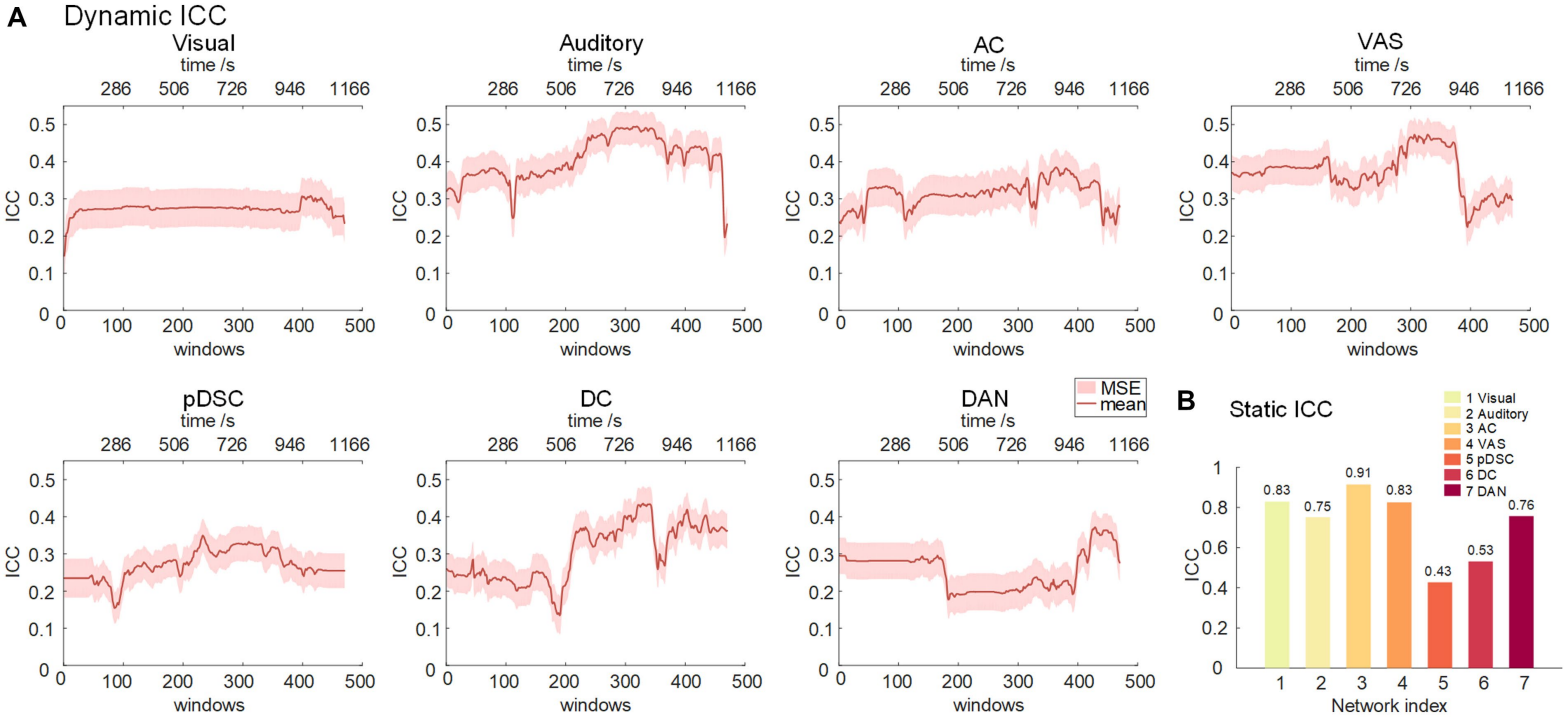
Intra-group correlation coefficient (ICC) for seven representative FBNs. **(A)** Dynamic ICC. The red line represents the group-wise dynamic ICC. **(B)** Static ICC.

It was assumed that similar sensory experiences would lead to the emergence of DFNs in a consistent and reliable manner. Therefore, we assessed the level of reliability of the DFNs that develop in response to the movie storyline across two sessions. Specifically, we first calculated the scan-wise intra-group correlation coefficient (ICC) values for seven static FBNs. The results showed that the visual, AC, and VAS networks exhibited excellent reliability, the auditory and DAN networks possessed good reliability levels, and the pDSC and DC networks had moderate reliability, indicating that the networks associated with primary perceptual processes were relatively more reliable, while the higher-level networks showed less reliability, consistent with previous studies (Choe et al., 2017) (Figure 6B).

We further analyzed the reliability of the DFNs (see Methods). The reliability of the auditory, AC, VAS, and DC networks, triggered by this touching movie, gradually increased during the mid to late period (about 300–390 window, corresponding to 600–1,000 s time period), and reached its peak in the near-end period. This is consistent with the narrative structure of the movie (Supplementary Table S2), wherein the plot also reaches its climax near the end (at around 17 min or 1,020 s) (Nguyen et al., 2017). The findings suggest that as the storyline develops, individuals may experience greater behavioral constraints and engagements, leading to an increase in the test–retest reliability of brain activities.

## 3. Discussion

Brain is a complex and dynamic system, composed of different brain regions forming functional brain networks that perform different cognitive functions (Raichle, 2006; Allen et al., 2014; Monti et al., 2014; Shine et al., 2016). This study explored the dynamic functional brain networks (DFNs) involved in higher-order cognitive processes, sensory perception, and emotional responses to naturalistic stimuli. Utilizing the proposed method, the study revealed rich and complex higher-order FBNs, including cerebellum-related networks, which are challenging to detect by conventional resting-state paradigm. The spatial patterns of these FBNs were time-varying with the movie storylines, and were correlated with the movie annotations and the subjective experience of the participants.

Specifically, our findings showed that two cerebellum-related networks, the DC network, and the pDSC network, were significantly connected to movie annotations. The DC network, which involves the cerebellum, posterior cingulate cortex, and precuneus region, was significantly and positively correlated with the appearance of positive facial expression during movie viewing. This finding is consistent with previous studies, which have shown that the cerebellum and posterior cingulate cortex are involved in facial emotion recognition, and that the precuneus is activated during the appearance of happy faces (Pelletier-Baldelli et al., 2015). The pDSC network, including partial DMN, salience network, and cerebellum, showed a significant positive correlation with the appearance of changepoints in movie scenes. This finding is also in line with previous studies, which have demonstrated that the changepoints in movie scenes are related to bottom-up attention, and that the salience network (SN) provides effective control of DMN activity when external event stimuli require an attentional response (Kelly et al., 2008; Menon and Uddin, 2010; Jilka et al., 2014). In addition, the cerebellum also plays a part in bottom-up attention (Gottwald et al., 2003; Kellermann et al., 2012). Overall, our results add weight to the notion that cerebellum-related DFNs are involved in higher-order cognitive and emotional processes.

Furthermore, the dynamic ISC analyses demonstrate relatively low consistency in the neural responses of higher-order brain networks across individuals. This variability suggests individual differences and unique experiences during natural viewing conditions, as evidenced by the strong correlations between the subjective ratings of the movie and dynamic ISC distances of DC and pDSC networks revealed by IS-RSA analyses. Exceptionally, the AC network exhibited relatively high temporal consistency across subjects but also existed a significant correlation with individuals’ subjective movie ratings, where the high ISC values were probably caused by the involvement of large auditory regions in the AC network. This could also explain the relatively weaker correlation between the AC network and movie ratings in comparison to that of the DC and pDSC networks (Figure 5 and Table 5). Intriguingly, all these three networks involve cerebellar posterior crus1,2 and vermis regions. Previous functional connectivity studies have confirmed that there are strong links between the posterior cerebellum and the temporal lobes, suggesting these regions share neural similarities and are involved in sensory integration and emotional processing (Yeo et al., 2011; Chan et al., 2019; Heleven et al., 2019; Van Overwalle et al., 2020b). In addition, several positron emission tomography (PET) studies suggest that the posterior cerebellum is involved in various emotional responses, such as fear, sadness, and happiness (Schwartz and Davidson, 1997; Turner et al., 2007; Verger et al., 2020). Additionally, Nguyen et al. (2017) have shown that the crus1,2 areas in the posterior cerebellum exhibit peak activities during important moments embedded in the movie, and Van Overwalle et al. (2020a) have shown that the cerebellar posterior crus 2 is specialized for mentalizing appraisal processes. Our study extends these previous findings by demonstrating that the dynamic nature of cerebellum-related FBNs is significantly correlated with individual-specific emotional responses.

Finally, our results also demonstrated that the DFNs elicited by ecologically valid sensory experiences were reproducible and reliable. Seven representative FBNs identified by our method were consistent across two scanning sessions with relatively-long interval (Figure 2, Supplementary Figure S1, and Table 1), and the changes in NVA and IVA for DFNs also showed high PCC values across two sessions, suggesting substantial consistency (Figures 2, 3, Supplementary Figures S2, S3, and Table 2). These results indicate that DFNs are reproducible during repeated movie viewing, further demonstrating that the naturalistic paradigm provides reliable experimental conditions for measuring DFNs. Furthermore, our results suggest that DFNs show good test–retest reliability, and the development of the movie plot enhances the test–retest reliability of the auditory, AC, VAS, and DC networks. This may reflect an increase in cognitive engagement as the storyline progresses, where the positive influences of cognitive participation on reliability appear to exceed the negative effect of familiarity from potential repeated viewings (Wang et al., 2017).

Overall, our study of time-varying spatial patterns of FBNs in the context of naturalistic paradigm improves our understanding of human perception, emotion, and subjective cognition. The results highlight the reliable correlations between cerebellum-related DFNs and sensory, cognitive, emotional, and subjective senses, which could motivate further research on the neural mechanisms underlying ecologically valid sensory experiences. Thus, our study provides valuable insights into the dynamic nature of brain networks and their role in higher-order cognitive and emotional processes, with potential applications in both basic and clinical neuroscience.

## 4. Limitations and future directions

Linking neural activity to higher cognitive and emotional functions in a dynamic and complex natural environment remains a challenge. In this work, we selected a relatively long time window of 60TRs to capture the accumulation of higher-order complex emotions and to improve the reproducibility of the FBNs (Savva et al., 2019). However, the relatively slow temporal resolution of fMRI with a large window size hinders the assessment of the responses of the brain to the perception of transient movie features. In the future, we expect to address this limitation by using electroencephalography (EEG) or magnetoencephalogram (MEG) with higher temporal resolution.

While the dataset used in this study is relatively small, all individuals watched a complete movie (20 min), which has been shown to strongly stimulate higher-order cognitions and emotions (Jaaskelainen et al., 2021). To increase the accuracy and reliability of our results, we performed a second acquisition after 3 months, despite the considerable expenses incurred for the acquisition of the complete movie. Nevertheless, we acknowledge that an abundance of subjects would further strengthen our findings, and we plan to apply our model to NfMRI datasets with a larger sample size in future studies.

## 5. Materials and methods

### 5.1. Experimental paradigm

The experiment consisted of two scanning sessions. Following a first session (session A) conducted 3 months earlier, participants underwent a second scanning session (session B) employing the same experimental paradigm. In each session, participants freely watched the 20-min short film “Butterfly Circus.” In addition, all participants completed a questionnaire immediately after session A.

The short film, “Butterfly Circus,” depicts a touching story of a limbless man who is encouraged by the showman of a renowned circus to discover his true potential. The narrative architecture of the film follows three distinctive drama acts that feature significant developments, complications, and turning points (Supplementary Table S2). Additionally, basic movie annotations were provided, including: the use of language, changepoints, the presence of positive valence of scenes, the presence of negative valence of scenes, the presence of faces with positive, and the presence of faces with negative (Supplementary Figure S6). Further details regarding the participants can be found in the Supplementary material (1.2).

### 5.2. Data acquisition and preprocessing

Sixteen right-handed (ages 27 ± 2.7) healthy participated in this study. FMRI images were acquired from a whole-body 3 T Siemens Trio MRI scanner with the following scanning parameters: repetition time (TR) 2,200 ms, echo time (TE) 30 ms, flip angle (FA) 79°, the field of view (FOV) 192 × 192 mm, pixel bandwidth 2,003 Hz, a 64 × 64 acquisition matrix, 44 axial slices, and 3 × 3 × 3 mm 3 voxel resolution. Functional images were preprocessed using FMRI Expert Analysis Tool (FEAT) from FMRIB’s Software Library (https://fsl.fmrib.ox.ac.uk/fsl/fslwiki), which involved motion correction, slice timing correction, spatial smoothing with 6-mm full width at half maximum Gaussian kernel, band pass filtering (0.0085 ± 0.15 Hz), linear registration to the standard Montreal Neurological Institute space (2 mm MNI152 standard template), and masking.

### 5.3. Dynamic sparse representation

**Figure 7:**
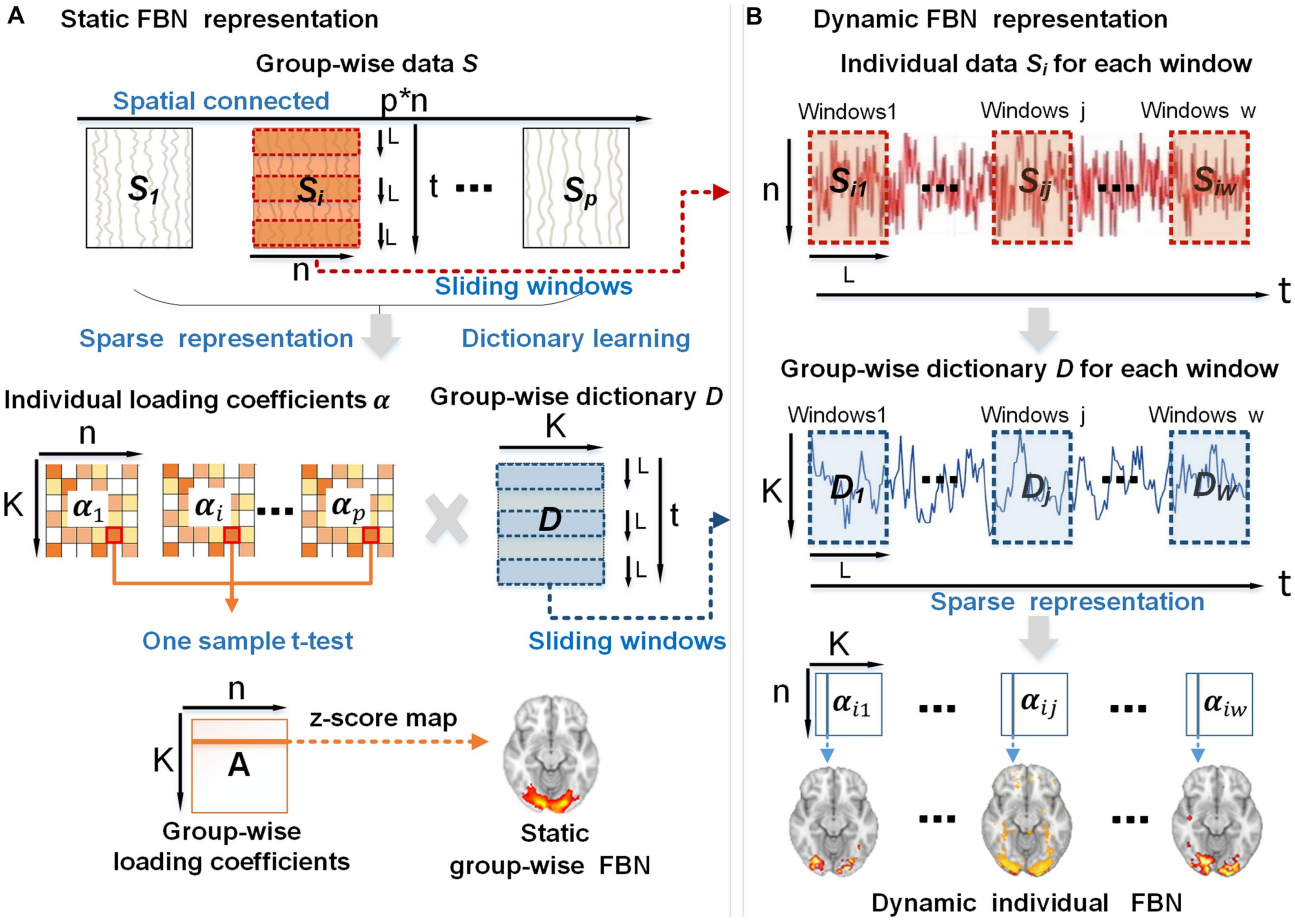
The overview of the proposed framework. **(A)** Using group-wise dictionary learning and sparse coding to represent static FBNs. **(B)** Sliding-window method applying for the representation of dynamic individual FBNs. *p*, the subject number; *n*, the number of voxels in the brain; *K*, the number of atoms in the dictionary; *t*, the time points; *L*, the length of each window; *w*, the total number of windows generated by the sliding-window method.

To discover and characterize DFNs, we proposed a computational framework comprised of two stages: (A) using group-wise dictionary learning and sparse coding (DLSC) to represent static FBNs (Figure 7A), (B) sliding-window method applying for the representation of dynamic spatial patterns of FBNs (Figure 7B).

In stage A, first, the whole-brain fMRI signals of each subject were extracted and stacked into a 2D matrix $S_{i}\;\left(S_{i}\in {\mathbb{R}}^{t\times n)}\right)$, followed by spatial concatenation of the individual matrix $S_{i}$ into a group-wise matrix 𝑺 $(S_{i}\in {\mathbb{R}}^{t\times \left(p\times n\right)})$ (top panel in Figure 7A), where t represents the time length of fMRI signals, n refers to the number of the individual brain voxels, p stands for the number of subjects. Second, we applied the dictionary learning algorithm to the group-wise matrix 𝑺 to learn a meaningful group-wise dictionary 𝑫 $(D\in {\mathbb{R}}^{t\times K})$. This dictionary 𝑫 consists of K atoms that can well represent the temporal features embedded in naturalistic fMRI data and are commonly shared by all the subjects (Ren et al., 2017a; Ge et al., 2020). Hence the group-wise dictionary 𝑫 can be used to sparsely represent individual fMRI signals $S_{i}$, resulting in the individual spatial patterns $\alpha_{i}\;\left(\alpha_{i}\in {\mathbb{R}}^{K\times n}\right)$ (K < <n) that exhibit correspondences across subjects (middle panel in Figure 7A). Notably, we used the online dictionary learning and sparse coding algorithm, which is an effective method for extracting instinctive information from the original signal and is suitable for group-level data operations (Ponce and Sapiro, 2010; Lv, 2013). Third, to obtain the group-wise static FBNs, we performed one-sample t-test on each element of individual loading coefficient matrix $\alpha_{i}$ (middle panel of Figure 7A). Specifically, for all the subjects together, we hypothesized that each element in the loading coefficient matrix $\alpha_{i}$ is group-wisely null. To evaluate this assumption, we conducted one-sample t-test on the corresponding element in the loading coefficient matrix $\alpha_{i}$ for different subjects, in order to test whether this hypothesis was accepted or rejected (Ren et al., 2017a). The resulting t-value was then transformed into a z-score, forming a group-wise loading coefficient matrix **A** composed of z-scores (Friston et al., 1994). Since each individual coefficient matrix $\alpha_{i}$ is sparse, the t-test result of the group-wise loading coefficient matrix **A** is also sparse. Afterwards, each row of matrix **A** can be mapped back to brain volume with z-scores, referred to as z-score maps. Consequently, the z-score map obtained from this analysis can depict spatially consistent activation across all subjects, thus representing the static group-wise FBNs (bottom panel of Figure 1A).

In stage B, in order to obtain a series of dynamic spatial patterns that evolve over time for each subject, we slid the same time window on the individual signal matrix $S_{i}$ and group-wise dictionary 𝑫 simultaneously. This approach not only allows us to establish correspondence of individual-level FBNs among individuals, but also generates the corresponding dynamic FBNs. Consequently, we obtained individual signal matrices $\;S_{i1},S_{i2},\ldots ,S_{ij},\ldots ,S_{iw}$, which represent the individual’s signal for each window, as well as group-wise dictionaries $\;D_{1},D_{2},\ldots ,D_{j},\ldots ,D_{w}$, which represent the group-wise dictionary for each window (top two panels in Figure 7B). The chosen window length, denoted as L (in our study, L = 60 TRs with a step size of 1 TR), resulted in a total of w = 470 windows. Consequently, the individual signal matrix $S_{i}\;$and group-wise dictionary 𝑫 were divided into multistage signal matrices $\;S_{ij}$ and dictionaries $\;D_{j}$ (j∈1, 2, …, w) (top two panels in Figure 7B). Next, based on $S_{ij}$ and $D_{j}$ of each window, we leveraged sparse representation algorithms to extract a sequence of coefficient matrices $\alpha_{ij}$ to represent individual dynamic spatial patterns of FBNs (bottom panel in Figure 7B). The above experiments were performed on each subject in session A and session B.

To identify the matching FBNs across two sessions, we manually selected representative FBNs from session A and match them with responding FBNs in session B based on their highest Pearson Correlation Coefficient (PCC) values and the maximum number of overlapping voxels (overlapping rate) (Benesty et al., 2009; Lv et al., 2015a). A detailed pipeline for selection of representative FBNs can be found in the Supplementary material (1.4). The PCC was defined as the correlation between the representative FBNs of session A and session B, and the overlapping rate of the FBNs in session A and session B was defined quantitatively as:


(1) $$R\;\left(X\mathrm{,Y}\right)=\frac{|X\cap Y|\;}{\left|Y\right|}$$

where 𝑿 is the representative FBN of session A, and Y refers to responding FBN of session B.

The DLSC algorithms rely on two key parameters: the number of dictionary atoms (*K*) and the sparsity penalty parameter (*λ*). However, there is no golden standard for determining the optimal values of these parameters. Based on previous studies that used DLSC algorithms for FBNs recognition, *K* was experimentally and empirically set to 400, and *λ* was set to a range of 0.1–0.5 (Lv et al., 2015b; Zhang et al., 2017; Ge et al., 2018). Therefore, in this study, we set *K* = 400, while systematically tested the setting of *λ* (0.1, 0.5). Through our experiments, we found that the largest number of networks could be identified with manual inspection when utilizing the parameters *K* = 400 and *λ* = 0.5. Consequently, we chose this set of parameters.

The window size is a crucial parameter that can determine the tradeoff between time resolution and estimation results. Previous related studies have empirically converged to window size values between 30 and 240 s (Hutchison et al., 2013; Preti et al., 2017). Additionally, Savva et al. (2019) suggested window size of at least 120 s to ensure the reproducibility of the result. Hence, we selected a window width of 60TRs (132 s).

### 5.4. Association between dynamic functional network and movie annotations

To quantify the dynamic changes of spatial patterns of FBNs, we employed two methods including calculating the number of activated voxels (NAV) and the intensity of activated voxels (IAV) of each FBN across all windows, respectively. Specifically, NAV was derived by counting the number of all voxels exceeding the threshold value (*z* = 1.65), while IAV was obtained by averaging the intensities of all voxels above this threshold. The group-wise NAV/IAV was derived by computing the average value of the NAV/IAV across all individuals.

The sliding window method produced 470 DFNs with a window length of 60 TRs and a step size of 1. Correspondingly, the duration of the scan was 530 TRs, with each TR corresponding to an annotation, resulting in 530 movie annotations in total. To establish correspondence between the DFNs and movie annotations, we selected the movie annotations occurring at the center point of each temporal window to correspond to each DFN based on previous studies (Simony et al., 2016; Tzachor and Hoshen, 2022). Specifically, we focused on a segment of movie annotations spanning from 31 to 500 TRs and examined their correlations with corresponding 470 dynamic FBNs.

The Spearman correlation coefficient between group-wise NAV/IAV and annotation vectors was computed to explore the association between group-wise dynamic changes in spatial patterns of FBNs and movie annotations, which were constructed for Language, Changepoint, Positive Scenes, Negative Scenes Positive Faces, and Negative Faces. Movie annotations were converted to vectors of 0 and 1 based on their onset and offset times (Supplementary Figure S6). To establish the statistical significance of the observed correlations, the correlation between the group-wise NAV/IAV and annotation vector was re-calculated 5,000 times by shuffling the vectors in each iteration. The observed correlation was compared with a null distribution of correlation generated by 5,000 permutations. If the observed correlation falls in the extreme tails of the distribution (i.e., the value of p is less than 0.05), we can conclude that there is a significant difference in group-wise NAV/IAV and movie annotation.

### 5.5. Dynamic inter-subject correlation

Inter-subject correlation (ISC) analysis measures the inter-subject consistency for temporal responses across participants (Hasson et al., 2004; Di and Biswal, 2020). To evaluate the ISC of dynamic FBNs, we first used the group-wise static FBNs as masks to extract the time-series signals of the corresponding FBN for each participant. Next, we averaged all the time-series signals within FBN, resulting in the FBN-level time-series signals for each FBN. Subsequently, we also applied the sliding window strategy and calculated the ISC of the FBN-level time-series signals in each time window for each subject, where the time window size was set to the same value as that in the “Dynamic sparse representation” section (i.e., 60TRs). Consequently, we derived the dynamic ISC metric for each subject for each representative FBN. To calculate the dynamic group-wise ISC metric, we performed Fisher z-transformation on the ISC values of all subjects for each window and subsequently averaged the ISC value across all individuals for each window.

### 5.6. Movie rating representation

The study employed a post-movie questionnaire to collect participants’ subjective appraisals of the movie, which consisted of eight questions. However, the RSA analysis excluded four questions because there was insufficient variability among participants (Supplementary Data). The remaining four questions are more focused on evaluating the movie subjectively, that is, how participants rated their feeling during the first movie session, including boredom, enjoyment, feeling happy or sad, and audio quality. Regarding question 4, specifically, the audio quality does not vary while recording, and each participant said they all had a comparable understanding of the movie’s plot. The participants’ level of engagement may have influenced how they rated the scale. All questions in the survey utilized a 1 to 5 rating scale. To represent participant differences in movie ratings, we employed a multidimensional scaling method to map responses to the questionnaire onto a two-dimensional representation.

### 5.7. The link between movie ratings and dynamic ISC

Inter-subject representational similarity analysis (IS-RSA) is a promising approach for examining the potential relationship between inter-subject variability in brain dynamics and individual differences in behavioral disposition (Kriegeskorte et al., 2008; Finn et al., 2020; Meer et al., 2020). Thus, we conducted the IS-RSA to assess the correlation between post-hoc behavioral movie ratings and dynamic ISC distances across all subjects.

We constructed inter-subject distance matrices to represent movie impressions and dynamic ISC. Specifically, inter-subject distances for movie impressions were calculated by measuring the Euclidean distance of questionnaire ratings between each possible pair of participants, resulting in 16 (number of participants) × 15 matrices. To examine the dynamics ISC distance, we calculated the Pearson distance between the dynamics ISC matrices for every possible pair of participants, producing a dynamic ISC distance matrix of size 16 × 15.

To assess the strength of associations between the movie ratings and dynamic ISC, we calculated the Spearman correlation between the lower triangular parts of the above two distance matrices. To assess the statistical significance of the results, we performed permutation testing 5,000 times. For each iteration, we squeezed the two matrices, dynamics ISC distance and movie rating distance, into row vectors, and randomly selected a new starting point for each row vector. This procedure allowed us to generate a null distribution of correlations and determine whether the observed correlation was significant.

### 5.8. Test–retest reliability of DFNs

To assess the level of reliability of dynamic FBNs during the natural viewing conditions, we calculated the test–retest reliability of the matching dynamic FBNs across two sessions. Specifically, we measured the intra-group correlation coefficient (ICC) for each window to determine the level of consistency in the FBNs across time (Shrout and Fleiss, 1979; McGraw and Wong, 1996). For comparison, we also calculated the static test–retest reliability of FBNs by calculating ICC over the entire period. ICC can be defined by the following equation:


(2) $$\mathrm{ICC}=\frac{{\mathrm{MS}}_{p}-{\mathrm{MS}}_{e}}{{\mathrm{MS}}_{p}+\left(d-1\right){\mathrm{MS}}_{e}}$$

Here, d refers to the number of observations, which in our study was equal to 2. ${\mathrm{MS}}_{p}$ represents the mean square variation between subjects, while ${\mathrm{MS}}_{e}$ represents the mean square variation within subjects. The test–retest reliability was divided into five levels: excellent (ICC > 0.8), good (ICC 0.6–0.79), moderate (ICC 0.4–0.59), fair (ICC 0.2–0.39), and poor (ICC < 0.2). The test–retest reliability was assessed at the scan-wise level, and the methodology for this process was carried out in accordance with the previous study (Guo et al., 2012; Wang et al., 2017).

## Data availability statement

The original contributions presented in the study are included in the article/Supplementary material, further inquiries can be directed to the corresponding author.

## Ethics statement

Ethical review and approval was not required for the study on human participants in accordance with the local legislation and institutional requirements. The patients/participants provided their written informed consent to participate in this study.

## Author contributions

LS and YR contributed to the conception and design of the study. LS, YR, and JN drafted the manuscript and performed the research. LS and KW contributed to analyzing the data. YH and XH contributed to supervision, writing review, and editing. All authors contributed to the article and approved the submitted version.

## Funding

This work was supported by the National Natural Science Foundation of China (Grant Nos. 62006187, 61971350, and 12271434), the Youth Innovation Team Foundation of Education Department of Shaanxi Province Government (Grant No. 21JP119), the China Postdoctoral Science Foundation Funded Project (Grant No. 2021M702650), the Natural Science Basic Research Program of Shaanxi (Grant No. 2023-JC-JQ-57), and the Key Research and Development Program Project of Shaanxi Province (Grant No. 2020SF-036).

## Conflict of interest

The authors declare that the research was conducted in the absence of any commercial or financial relationships that could be construed as a potential conflict of interest.

## Publisher’s note

All claims expressed in this article are solely those of the authors and do not necessarily represent those of their affiliated organizations, or those of the publisher, the editors and the reviewers. Any product that may be evaluated in this article, or claim that may be made by its manufacturer, is not guaranteed or endorsed by the publisher.
