# Supplementary material for: Mapping the time-varying functional brain networks in response to naturalistic movie stimuli
Source: Front Neurosci. 2023 Jun 16;17:1199150. doi: 10.3389/fnins.2023.1199150 (PMC10311647; doi:10.3389/fnins.2023.1199150)
Supplement: Supplementary file 1 [file Data_Sheet_1.docx]

Supplementary Material

**Mapping the Time-Varying Functional Brain Networks in Response to Naturalistic Movie Stimuli**

**Limei Song^1†^,** **Yudan Ren^1†*^, Kexin Wang^1^, Yuqing Hou^1^, Jingsi Nie^2^, Xiaowei He^1*^**

*** Correspondence:** Yudan Ren*: [yudan.ren@nwu.edu.cn](mailto:yudan.ren@nwu.edu.cn)

# Supplementary Data

## Movie Details

The Butterfly Circus is a short movie set in the 1930’s depression-era rural USA. The movie can be viewed at: [https://www.youtube.com/watch?v=y_MCwlY6zzg&t=6s]. Further details of the movie can be found on the Internet movie database (IMDB) [https://www.imdb.com/title/tt1507355/] and on Wikipedia: [https://en.wikipedia.org/wiki/The_Butterfly_Circus].

## Participants Details

The dataset utilized in our research was collected by the University of Queensland and originated from participants in English-speaking countries. The study involved 21 healthy participants, of which 11 were female, with ages ranging from 21 to 31 years (mean age 27 ± 2.7 years). All participants provided written informed consent. Ethical guidelines set by the National Health and Medical Research Council were followed, and the study was approved by the human ethics research committee of the University of Queensland.

To ensure a controlled viewing experience, participants confirmed that they had not previously seen the movie and were instructed not to watch it outside of the scanning sessions until the study's conclusion. The movie stimulus was presented using Presentation software from NeuroBehavioral Systems (USA) and displayed on an MRI-compatible monitor positioned at the rear of the scanner. The soundtrack of the movie was delivered through an MRI-compatible audio headphone from Nordic NeuroLab (Norway). From the initial 21 participants, five were excluded from the study. Three participants were excluded due to in-scanner excessive head motion, while the remaining two participants were excluded because complete data for their second scanning session were not obtained.

## The post-movie questionnaire

The post-movie questionnaire had eight questions Four questions assessed the following: whether participants had seen the movie before (none had), whether English was their native language (all participants), how participants rated their fluency in English (two participants indicated their fluency as a 4, while the rest indicated a 5.)

Two participants indicated their English fluency as a 4, while the rest indicated a 5. 5), and how well the participants understood the content of the movie (all participants indicated a 100% comprehension) (100% comprehension). These questions were omitted from the RSA analysis due to the lack of variability across participants. The remaining four questions were closer to closely assessed subjective evaluations of the movies and were used in the RSA.

1. were you bored during your first viewing of the movie?

Not at all: 1 2 3 4 5: Very bored

2. 2. How much did you enjoy the first movie?

Not at all: 1 2 3 4 5: Very much

3. How emotional were you during the first movie?

Very sad: 1 2 3 4 5: Very happy

4. How was the audio quality of the first movie?

Very poor: 1 2 3 4 5: Very good

## The pipeline for selection of representative FBNs

Initially, we identified activated voxels with activation value surpassing a predetermined threshold (1.65 in our work) in the group-wise spatial patterns from group-wise loading coefficient matrix **A**. We then utilized a criterion where spatial patterns with a higher count of activated voxels were considered more likely to represent networks. Thus, we focused on spatial patterns containing more than 20,000 activated voxels (Supplemental Fig. 7). This selection procedure yielded more than 100 spatial patterns.

Subsequently, we conducted manual inspections of these selected spatial patterns. Specifically, we compared our derived spatial patterns with the resting-state network (RSN) 10 template and other well-established resting-state networks, such as Yeo network (Smith et al., 2009; Yeo et al., 2011). Through this process, we identified classic networks such as the visual network, audio networks, and the dorsal attention network (DAN) in our work (Supplemental Figure 8a, b, g).

Additionally, we compared identified spatial patterns with FBNs previously identified under naturalistic stimuli (Ren et al., 2017). Through this comparison, we discovered the default mode network (DMN) and the cerebellar network (DC) which combines DMN and cerebellar network (Supplemental Figure 8f).

Furthermore, we encountered networks that were less commonly observed in traditional resting-state stimuli but were composed of combinations of well-known networks. For example, we discovered the auditory and cerebellar network (AC) network, which encompassed the auditory and cerebellar networks (Supplemental Figure 8c). Additionally, we detected a network termed VAS, consisting of the sensorimotor, visual, and audio networks (Supplemental Figure 8d). Moreover, we identified the partial DMN, the salience network, and the cerebellar network, collectively forming the pDSC network (Supplemental Figure 8e). These networks exhibited variations from conventional resting-state networks, likely due to the utilization of a naturalistic paradigm involving rich multimodal dynamic stimuli. This paradigm resulted in more complex patterns of functional brain activity and a greater diversity of FBNs (Saarimaki, 2021; Sonkusare et al., 2019).

During the manual inspection process, we identified a total of 22 networks for session A and 31 networks for session B. we further assessed the consistency of these identified networks obtained from the two sessions as an additional criterion. Specifically, we focused on identifying networks that consistently appeared in both sessions, based on their maximum number of overlapping voxels (overlapping rate) and the highest Pearson Correlation Coefficient (PCC) values (Tab. 1). This analysis provided confirmation for the inclusion of the seven networks discussed in our manuscript.

# Supplementary Figures and Tables

## Supplementary Figures


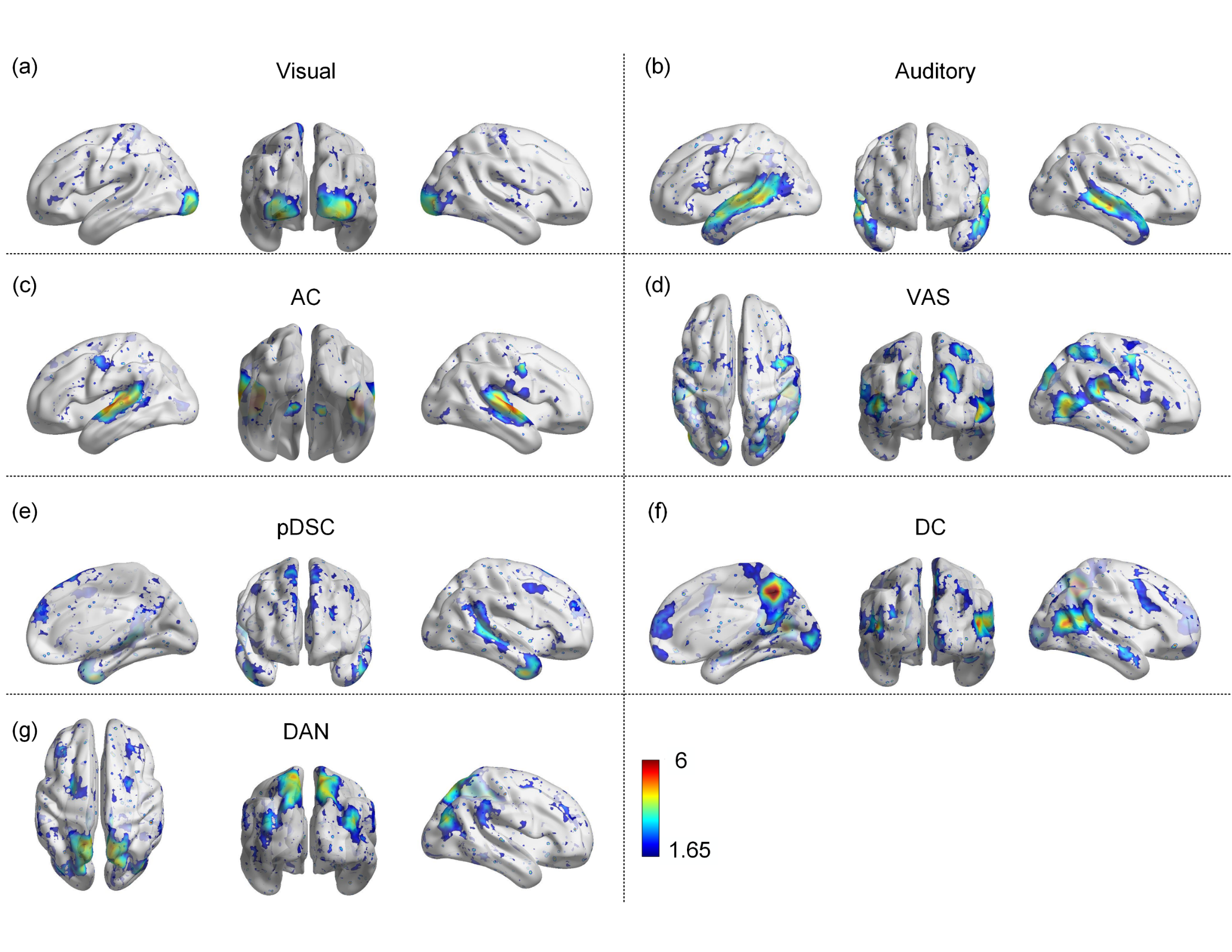


**Supplementary Figure 1**. Group-wise static functional brain networks (FBNs) of session B. (a) visual network, (b) auditory network, (c) auditory and cerebellar network (AC), (d) audiovisual and sensorimotor network (VAS), (e) partial default mode network (DMN), salience, and cerebellar network (pDSC), (f) DMN and cerebellar network (DC), (g) dorsal attention network (DAN).


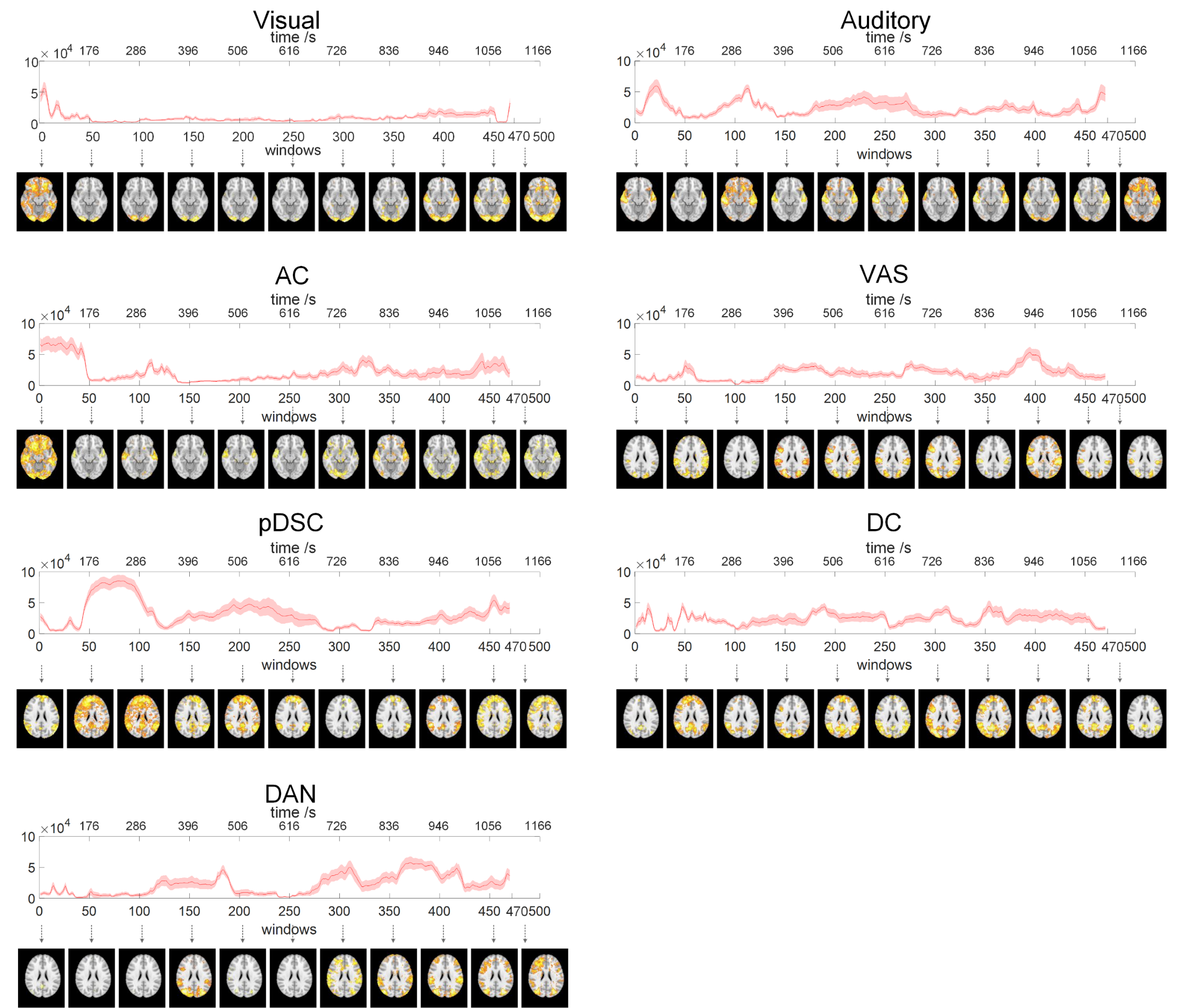


**Supplemental Figure 2**. Dynamic evolution of the number of activated voxels (NAV) of seven brain function networks (FBNs) (session B), The corresponding FBNs of the first window among every 50 windows are displayed at the bottom.


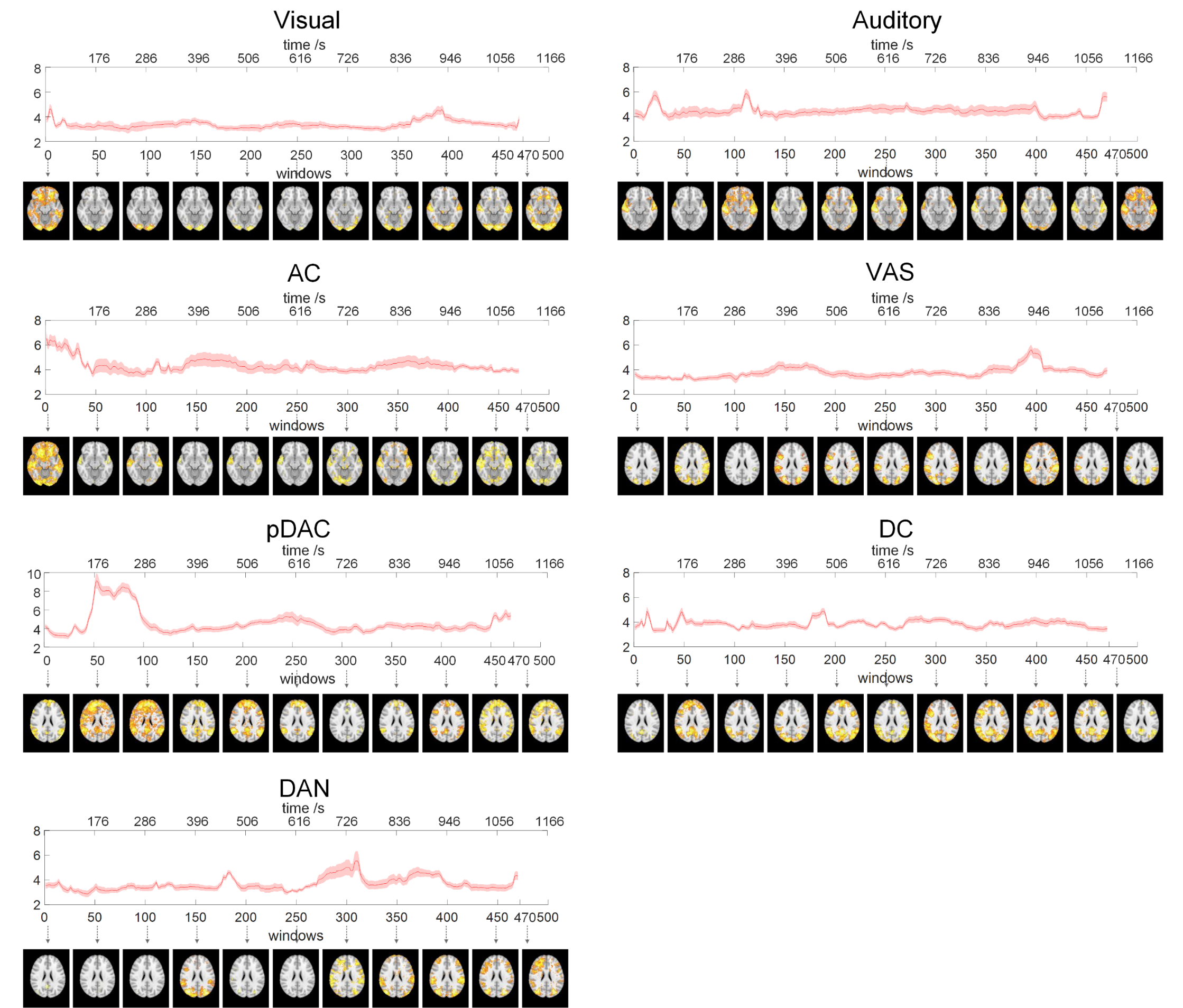


**Supplemental Figure 3**. Dynamic evolution of the intensity of activated voxels (IAV) of seven FBNs (session B), The corresponding FBNs of the first window among every 50 windows are displayed at the bottom.


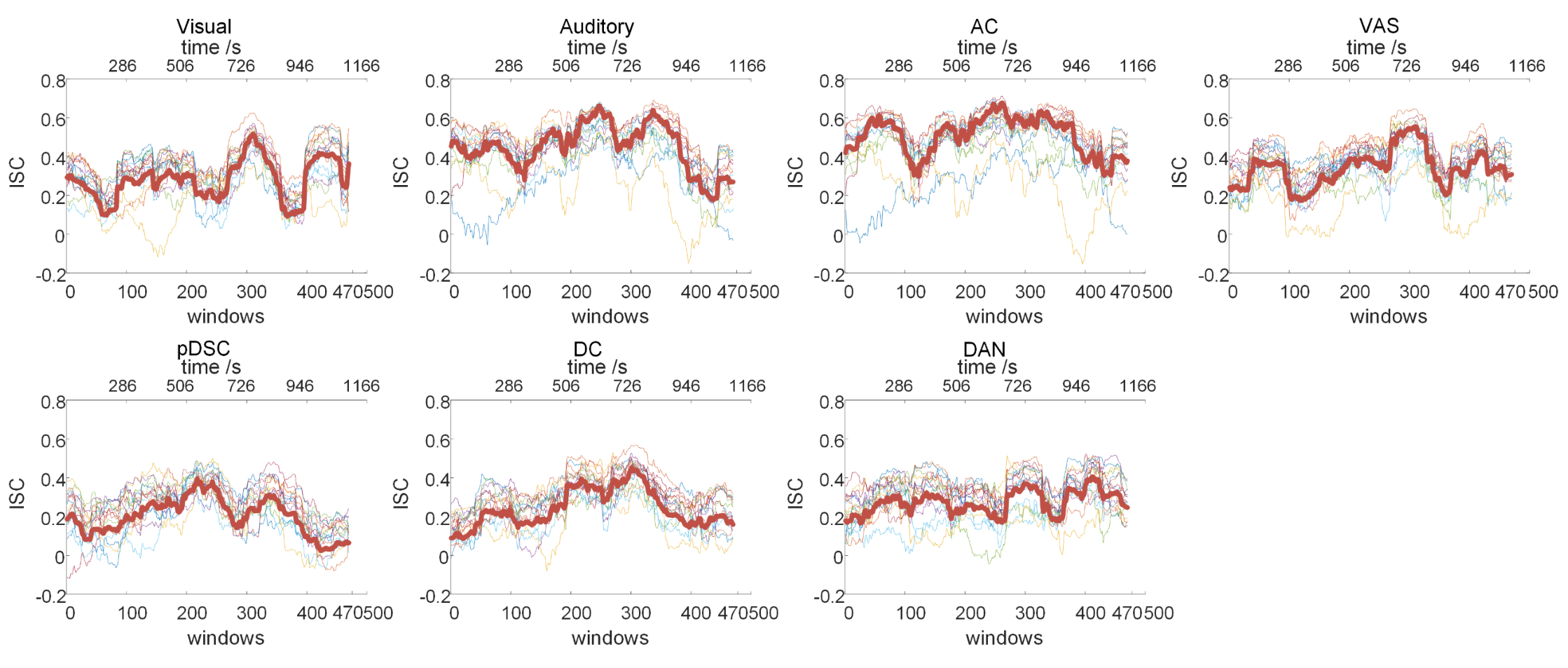


**Supplemental Figure 4.** Dynamic inter-subject correlation (ISC) (session B): Group-wise and individual dynamic ISC. The thick red line represents the group-wise dynamic ISC, and the thin colorful lines represent the dynamic ISC of 16 different individuals.


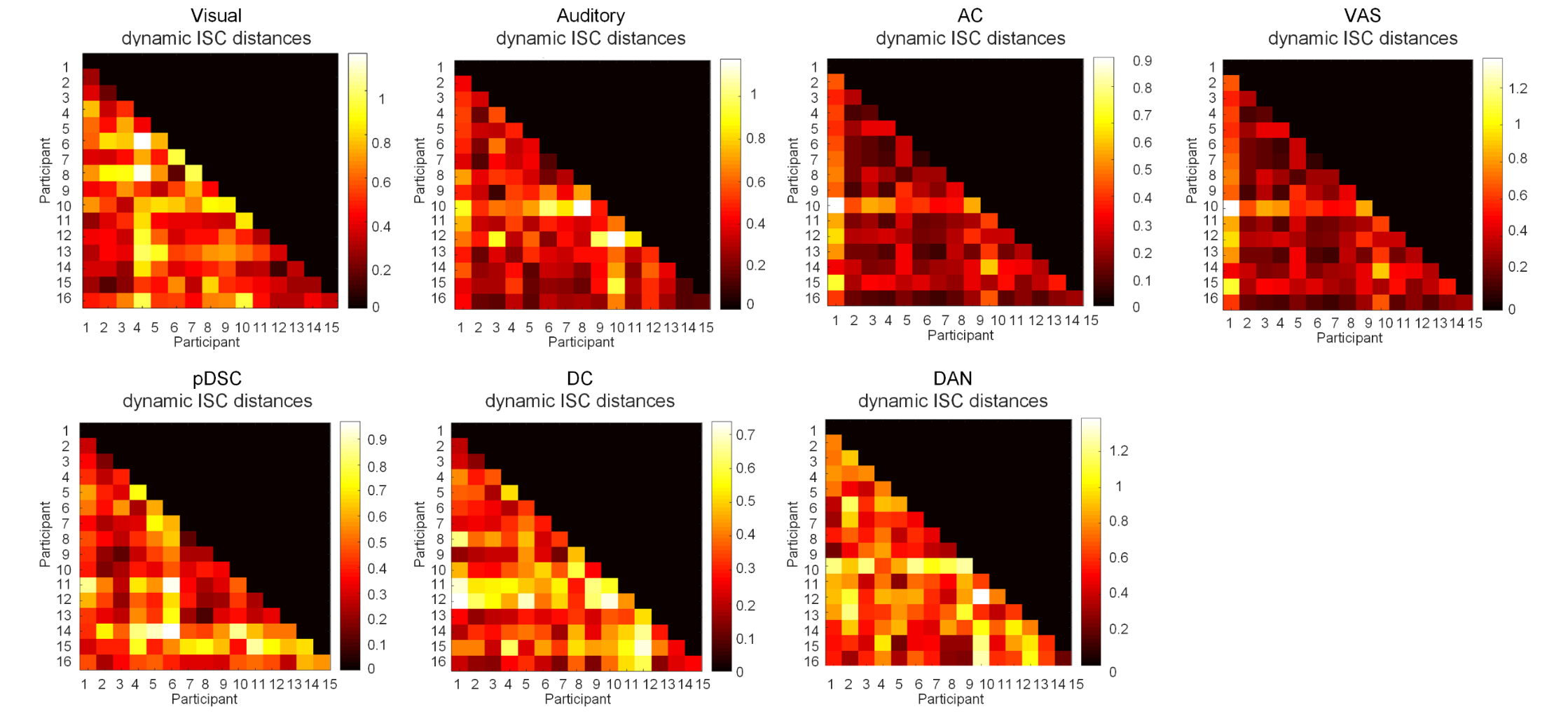


**Supplemental Figure 5.** The distance matrices of dynamic ISC for seven representative FBNs.


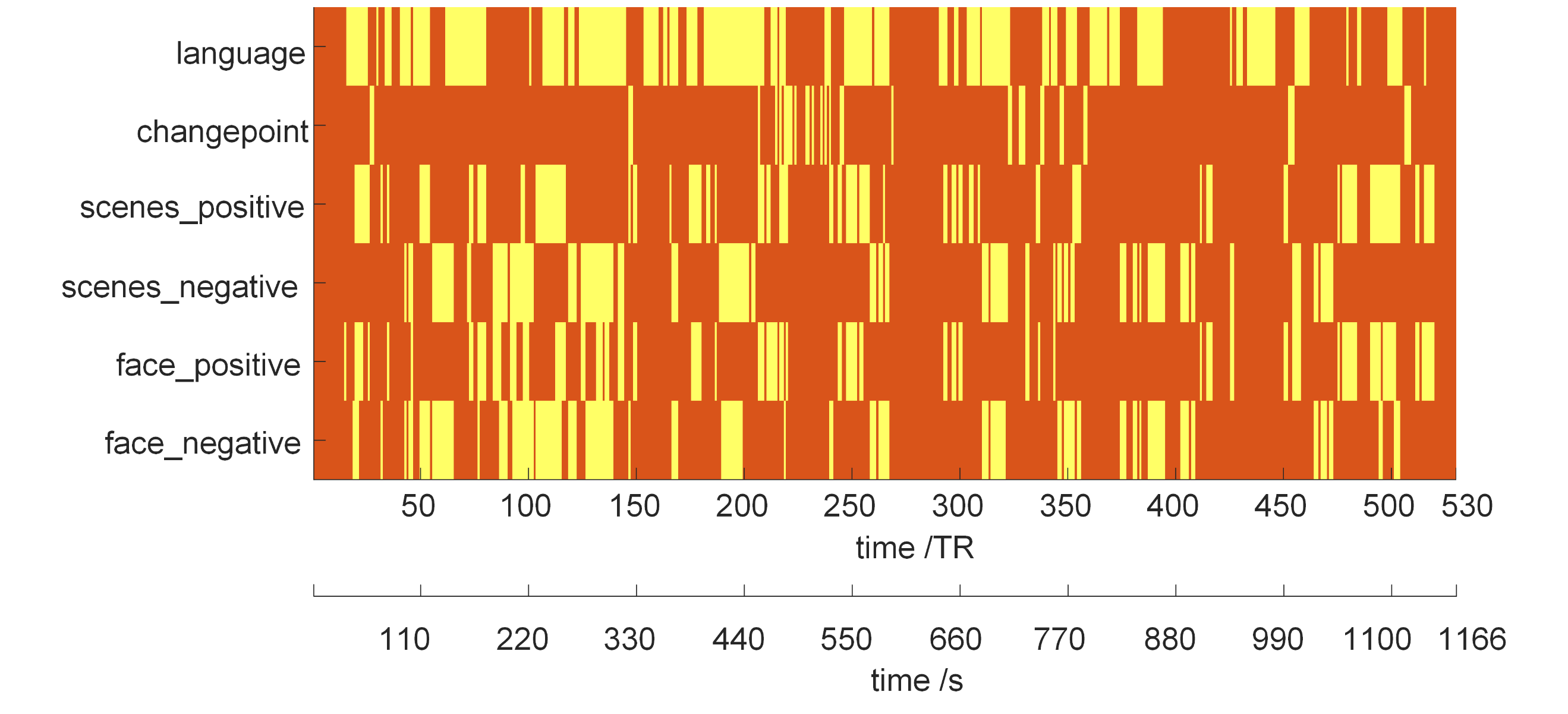


**Supplemental Figure 6.** Movie annotations. use of language, changepoints (a transition from one movie scene to another), occurrence of positive/negative faces, and positive/negative scenes. The time scales are in scans (one repetition time (TR) = 2.2 seconds), in seconds to facilitate inspection of the movie with a movie player. Movie annotations was manually and computer-assisted by human experts.


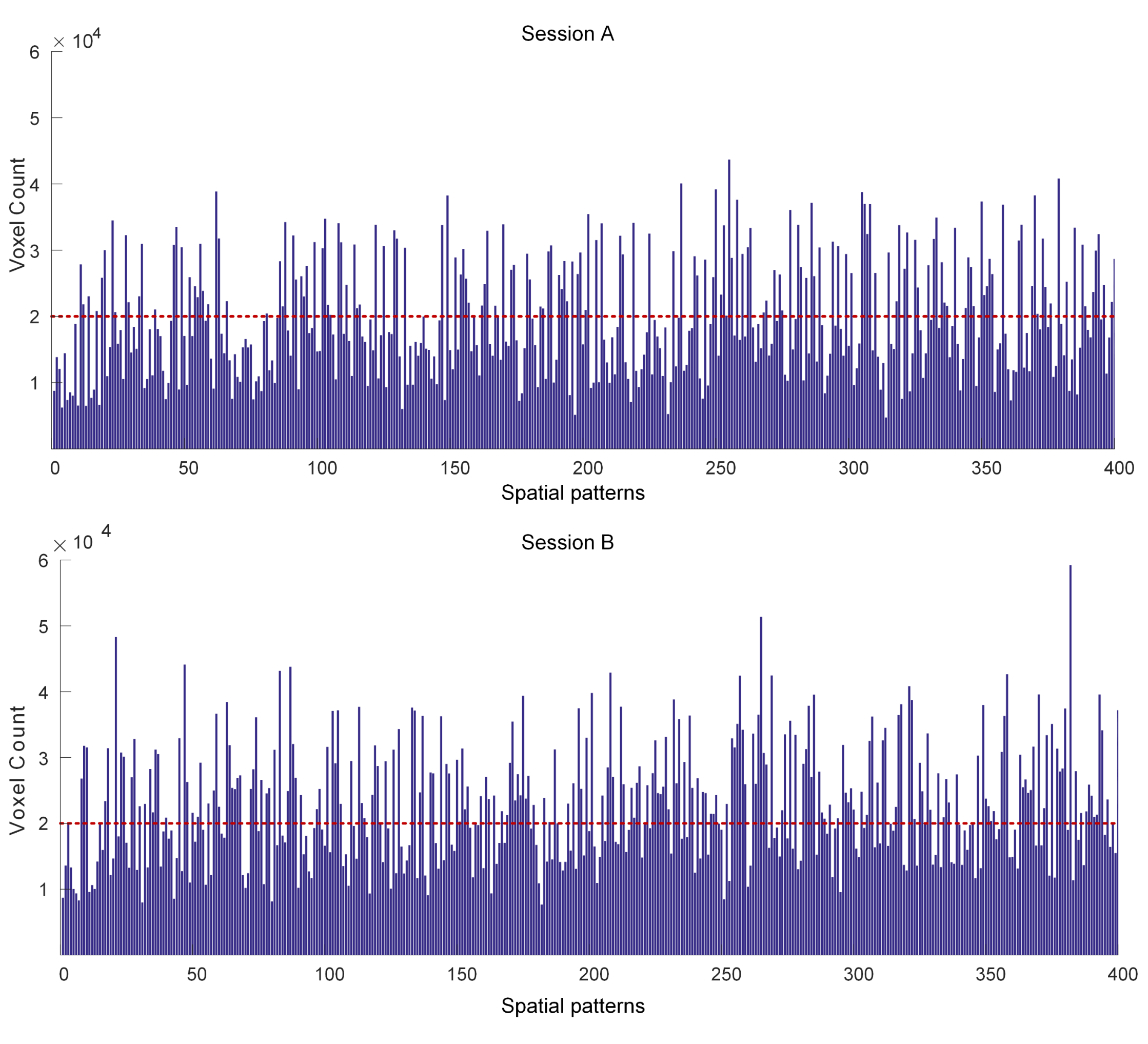


**Supplemental Figure 7.** Voxel activation exceeding the threshold (1.65) across 400 spatial patterns in two sessions.


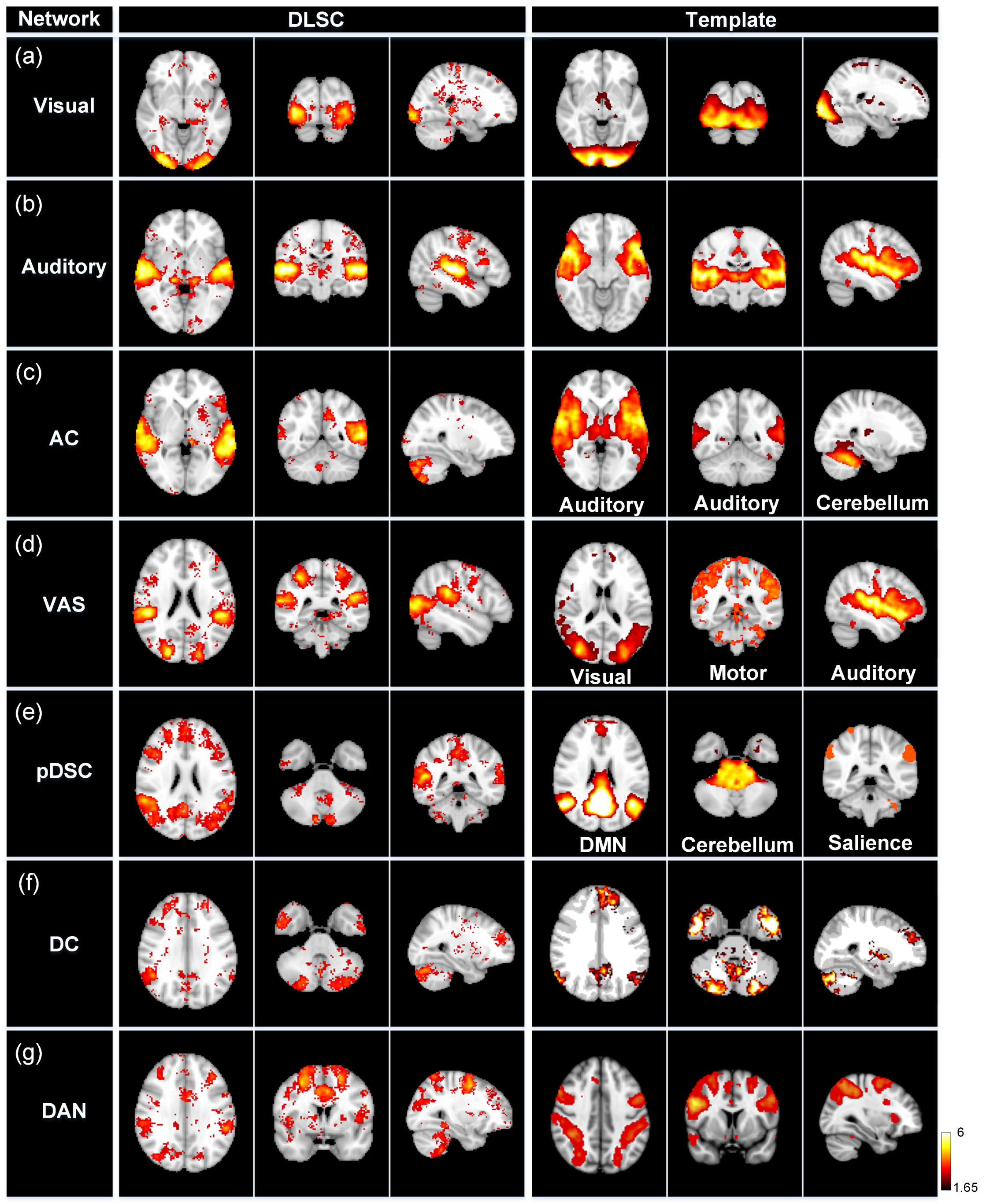


**Supplemental Figure 8.** Comparison of FBNs derived by the proposed dictionary learning and sparse coding (DLSC) method and the well-established resting-state template or previously identified networks under naturalistic stimuli. Note that the template columns in rows (c), (d) and (e) consist of two or three independent networks or templates.

## Supplementary Tables

**Supplemental Table1.** Pearson correlation between session A and session B for the number of activated voxels (NAV) and intensity of activated voxels (IAV).

|  | **Visual** | **Auditory** | **AC** | **VAS** | **pDSC** | **DC** | **DAN** |
| --- | --- | --- | --- | --- | --- | --- | --- |
| NAV | 0.76 | 0.96 | 0.76 | 0.78 | 0.20 | 0.57 | 0.89 |
| IAV | 0.83 | 0.92 | 0.55 | 0.92 | 0.11 | 0.74 | 0.62 |

Supplementary Table 2. Timing and description of the narrative architecture in The Butterfly Circus.

| **Time (min: sec)** | **Plot Point** | **Scene description** |
| --- | --- | --- |
| **Act One** | | |
| 04:04 | Catalyst: An inciting incident necessitates a major change for the protagonist | Will (the limbless man) is abused and humiliated at the sideshow, making him want to leave the carnival. Mendez (the Butterfly Circus showman) intervenes to help. The audience has a close view of Mendez approaching Will and Will’s fearful and aversive reaction as the handheld camera executes a whip pan, pushing in to a close-up of Mendez as Will recoils. |
| 04:11 | Catalyst | Mendez calls Will “magnificent” but his actions and words are intrusive and the unexpectedly intimate approach is uninvited so Will recoils and spits at him, fearing ridicule. A medium close-up of Will, shot over Mendez’s shoulder reveals the emotional force of this encounter. |
| 04:37 | Catalyst | An ugly close-up of the sideshow attendant shows him insulting Will, calling him a gimp. A close-up reaction shot of Will reveals that he is humiliated and angry. |
| 04:50 | Turning Point: establishes goal | Will realizes that he has just sabotaged his dream of escaping the sideshow and “joining one of those fancy shows.” |
| 05:03 | Turning Point | Tattooed man: “You just spat on the showman from The Butterfly Circus. That was Mendez…” |
| 05:14 | Turning Point establishes narrative question | A tattooed sideshow worker laughs cruelly at Will and we observe Will’s reaction in close-up as he realizes his predicament. This is an emotional low that establishes Will’s goal. This is also the point at which the narrative question is formulated: How can Will achieve his goal of improving his life by joining a better circus show? |
| **Act Two** | | |
| 06:20 | Development | The Butterfly Circus Strongman is startled when he discovers Will has stowed away in the truck. The audience sees a high angle mid-shot of Will lying in the truck with his back to the camera. Cheery background music plays on a gramophone when Mendez welcomes Will. |
| 06:50 | Development | Will meets a young boy called Sammy, who asks: “Where are your arms and legs?” and “will you be joining our show?” In this shot-reverse-shot dialogue sequence the camera looks down on Will slightly. |
| 07:07 | Obstacle (external) | Will discovers that he cannot attain his goal of joining a “fancy show” because The Butterfly Circus does not have a sideshow and Will has no skills to perform his own act. |
| 07:18 | Obstacle (external) | A low angle shot looking up at Mendez lends him authority as he asks Will why people visit sideshows. A close-up looking down on Will’s face shows that he understands the cruelty of such shows and that his goal of being in such a show is problematic. |
| 09:38 | Obstacle (internal) | An African-American boy admires the Strongman’s muscles and asks Will if he is also in the Butterfly Circus. |
| 09:40 | Obstacle (internal) | Will responds to the boy sadly, “No, not exactly”. This establishes an internal obstacle that Will must overcome: he feels unworthy and useless. |
| 09:56 | Obstacle (internal) | Walking away the boy says he wants to be just like the Strongman; his father tells him that he can do anything if he wants to enough. Will’s face reveals that he does not believe he can achieve greatness and be admired. |
| 11:35 | Turning Point: confronts internal obstacle | Mendez repeats humiliating taunts from the sideshow where Will used to work. Will is hurt and shocked, asking: “Why would you say that?” |
| 11:50 |  | Mendez: “Because you believe it.” This emotional low point when Will faces his internal obstacle is accompanied by harsh dialogue, raised voices, close-ups of faces with tearful eyes and soft, sad violin music. |
| 13:05 | Turning Point: establishes new goal | Mendez: “You have an advantage. The greater the struggle, the more glorious the triumph.” Mendez is framed in a low-angle close-up that privileges him in the frame. Will’s new goal is to find his talent and triumph over adversity. |
| 14:10 | Confronts Internal Obstacle | Will falls while trying to cross the river and he feels helpless. As Will shouts for help his circus friends in the distance can’t hear him over the sound of the river. |
| 14:20 | Confronts Internal Obstacle | Mendez walks by. Will asks Mendez for help but Mendez says, “I think you’ll manage.” Melancholy violin music underscores this low point and the camera is also at ground level, showing Will helpless on the ground. |
| 14:51 | Overcomes Internal Obstacle | Will starts struggling to get up by himself, with determination. A hand-held shot from Will’s perspective shows him figuring out how to climb onto the log. |
| 15:10 | Overcomes Internal Obstacle | Will stands up on the log and cheers triumphantly, then begins to cross the river to the tune of upbeat fiddle and tambourine music. |
| 15:34 | Confronts External Obstacle | Will falls off the log into deep water. It looks as though Will is drowning. The audience hears muffled underwater bubble sounds and imagery as the music takes on a serious tone with a high anxious note and a low bowing sound. |
| 15:46 | Confronts External Obstacle | The circus folk notice Will is missing and search frantically for him in the water. Shots from the perspective of Will’s circus friends show the surface of the water dark and still. |
| 16:20 | Overcomes External Obstacle | Will overcomes his external obstacle and bobs to the surface: “Look! I can swim!” |
| **Act Three** | | |
| 17:05 | Climax | Mendez tells the audience that Will is climbing 50 feet into the air and he will leap from a high platform and dive into a tiny water tank far below. Bird’s eye shot from Will’s point of view looking down at water from high diving board. |
| 17:26 | Climax | Will dives in and the crowd gasps. |
| 17:37 | Climax | Will then swims to the surface. Slow motion when Will surfaces and smiles. |
| 17:38 | Climax | Triumphant music and clapping. |
| 18:11 | Resolution | A disabled boy approaches Will after the show and hugs him. The boy’s mother thanks him for being inspirational. From the perspective of the boy, the camera looks up to Will as the boy embraces him and he smiles, having found his talent and attained a sense of self-worth. |

# Reference

Ren, Y., Lv, J., Guo, L., Fang, J., and Guo, C.C. (2017). Sparse coding reveals greater functional connectivity in female brains during naturalistic emotional experience. PLoS One *12*, e0190097.

Saarimaki, H. (2021). Naturalistic Stimuli in Affective Neuroimaging: A Review. Front Hum Neurosci *15*, 675068.

Smith, S.M., Fox, P.T., Miller, K.L., Glahn, D.C., Fox, P.M., Mackay, C.E., Filippini, N., Watkins, K.E., Toro, R., and Laird, A.R. (2009). Correspondence of the brain's functional architecture during activation and rest. Proceedings of the national academy of sciences *106*, 13040-13045.

Sonkusare, S., Breakspear, M., and Guo, C. (2019). Naturalistic Stimuli in Neuroscience: Critically Acclaimed. Trends Cogn Sci *23*, 699-714.

Yeo, B.T., Krienen, F.M., Sepulcre, J., Sabuncu, M.R., Lashkari, D., Hollinshead, M., Roffman, J.L., Smoller, J.W., Zollei, L., Polimeni, J.R.*, et al.* (2011). The organization of the human cerebral cortex estimated by intrinsic functional connectivity. J Neurophysiol *106*, 1125-1165.
